# Oligomycins inhibit *Magnaporthe oryzae Triticum* and suppress wheat blast disease

**DOI:** 10.1371/journal.pone.0233665

**Published:** 2020-08-17

**Authors:** Moutoshi Chakraborty, Nur Uddin Mahmud, Abu Naim Md. Muzahid, S. M. Fajle Rabby, Tofazzal Islam

**Affiliations:** Institute of Biotechnology and Genetic Engineering, Bangabandhu Sheikh Mujibur Rahman Agricultural University, Gazipur, Bangladesh; University of Nebraska-Lincoln, UNITED STATES

## Abstract

Oligomycins are macrolide antibiotics, produced by *Streptomyces* spp. that show antagonistic effects against several microorganisms such as bacteria, fungi, nematodes and the oomycete *Plasmopara viticola*. Conidiogenesis, germination of conidia and formation of appressoria are determining factors pertaining to pathogenicity and successful diseases cycles of filamentous fungal phytopathogens. The goal of this research was to evaluate the in vitro suppressive effects of two oligomycins, oligomycin B and F along with a commercial fungicide Nativo® 75WG on hyphal growth, conidiogenesis, conidial germination, and appressorial formation of the wheat blast fungus, *Magnaporthe oryzae Triticum* (MoT) pathotype. We also determined the efficacy of these two oligomycins and the fungicide product *in vivo* in suppressing wheat blast with a detached leaf assay. Both oligomycins suppressed the growth of MoT mycelium in a dose dependent manner. Between the two natural products, oligomycin F provided higher inhibition of MoT hyphal growth compared to oligomycin B with a minimum inhibitory concentration of 0.005 and 0.05 μg/disk, respectively. The application of the compounds completely halted conidial formation of the MoT mycelium in agar medium. Further bioassays showed that these compounds significantly inhibited MoT conidia germination and induced lysis. The compounds also caused abnormal germ tube formation and suppressed appressorial formation of germinated spores. Interestingly, the application of these macrolides significantly inhibited wheat blast on detached leaves of wheat. This is the first report on the inhibition of mycelial growth, conidiogenesis, germination of conidia, deleterious morphological changes in germinated conidia, and suppression of blast disease of wheat by oligomycins from *Streptomyces* spp. Further study is needed to unravel the precise mode of action of these natural compounds and consider them as biopesticides for controlling wheat blast.

## Introduction

Oligomycins are macrolide antibiotics, produced by some strains of *Streptomyces*. They have broad-spectrum biological activities against organisms like fungi, bacteria, nematodes and the oomycete *Plasmopara viticola* [1–4]. *Streptomyces* species are common soil-dwelling bacteria that have been broadly used as bio-control agents [5]. *Streptomyces* species produce a number of bioactive compounds possessing antifungal, antiviral, antibacterial, anticancer, nematicidal, and antioxidant properties [5, 6]. Several previous studies showed that the effectiveness of some strains of *Streptomyces* in biological control of phytopathogens largely depends on the production of oligomycins [7]. The oligomycins are mitochondrial F1F0 ATP synthase inhibitors that cause apoptosis in a number of cell types [8]. The oligomycin complex, which was first documented in 1954 in a strain of a soil bacterium, *Streptomyces diastatochromogenes* was highly inhibitory against fungi [1]. Antifungal, antitumor, insecticidal, immunosuppressive and nematicidal properties of oligomycins have also been reported [1–3, 7, 9]. The oligomycins contain analog isomers A through G that are highly selective for disrupting mitochondrial metabolism [3, 4, 8, 10]. Although biological activities of oligomycins on fungi and the oomycete *P*. *viticola* have been reported, very little is known about the effect of these natural products on the notorious wheat blast fungus *Magnaporthe oryzae Triticum* (MoT). The bioactivities of oligomycins against different classes of fungal species indicates that their targets may involve a variety of cellular processes, such as inhibition of mycelial growth of *Cladosporium cucumerinum*, *Magnaporthe grisea*, *Colletotrichum lagenarium*, *Botrytis cinerea*, *Cylindrocarpon destructans*, *Fusarium culmorum*, *Erysiphe graminis* and *Phytophthora capsici* [3, 11], lysis and motility inhibition of *P*. *viticola*, and *Aphanomyces cochlioides* zoospores [4].

The wheat blast fungus MoT is one of the most destructive pathogens of wheat [12–15]. The three-celled, hyaline and pyriform fungal conidium attaches to the host surface by secreted adhesive [14, 16, 17]. The attached conidium germinates to form a hyphal germ tube, an appressorium and a penetration peg to penetrate the epidermis of the host and complete the infection process [16, 18]. The invasion of plant tissue is achieved by penetrating the epidermal cells and invaginating the host plasma membrane [16–18]. The fungus can attack wheat plants at any stage of development and infects leaves, nodes, stems, and spikelets [15, 17, 19]. Mycelium can survive in the embryo, endosperm, and kernal tissues of wheat seed. Wheat blast mainly affects wheat heads; it bleaches the infected heads, resulting in deformed seed or no seed production [14]. The badly affected wheat heads can die, leading to a drastic reduction in grain yield. Bleaching of the spikelets or the entire head at an early stage is the most common recognizable symptom of the disease [12, 14, 15]. Infected seeds and airborne conidia usually disseminate the fungus which may survive in infected seeds and crop residues [20]. Pyriform conidia developed from conidiophores and conidia germination with appressorial development at the germ tube tips are essential steps of the disease cycle of MoT [16]. Disruption of any of these asexual life stages reduces the chance of pathogenesis and development of an epidemic [21]. Finding natural bioactive compounds capable of inhibiting any of these asexual life stages is considered the first step in the development of a new fungicide for controlling MoT.

Wheat blast was first found in Brazil in 1985, and subsequently spread to neighboring Bolivia and Paraguay [12, 19]. The cultivation of wheat (*Triticum aestivum*) has increased in Bangladesh in recent years making it the 2^nd^ largest food source after rice. A sudden outbreak of wheat blast occurred in Bangladesh in 2016, which was the first incident of wheat blast outside of South America [14, 22]. About 15,000 hectares of wheat were destroyed, resulting in about 15% crop losses in Bangladesh [14]. The outbreak concerned crop scientists as it has the potential to extend further to major wheat-producing regions in neighboring South Asian countries and Africa due to similar climatic conditions [23]. Plant pathologists have cautioned that this disease is expected to disperse to India, Pakistan and China, that rank 2^nd^, 8^th^ and 1^st^, respectively, in the world for wheat production, [15, 24].

Current wheat blast disease management methods include the utilization of synthetic fungicides. Natural products generally impart less harmful effects on the environment and health of living species including humans when compare to their synthetic contemporaries [25, 26]. Indiscriminate application of synthetic commercial fungicides for plant protection may also result in development of resistance in fungal population to fungicides [13, 17]. In Brazil and other South American countries, some MoT strains have developed resistance to strobilurin (QoI) and triazole fungicides [27, 28]. Nowadays, natural products that are environment-friendly with minimum toxicity to living organisms are gaining popularity as important ecologically suited alternative fungicides for protecting plants. Therefore, search for novel bioactive natural products against MoT is an urgently needed scientific endeavor.

The biological approach for plant disease management offers a better alternative to the control of wheat blast. There is very little information available on the antagonistic effects of *Streptomyces* spp., and/or secondary metabolites derived from them to control wheat blast. We screened 150 natural compounds belonging to the classes alkaloids, terpenoids, macrolides, macrotetrolides, tepenoids, and phenolics isolated from different plants and microorganisms for antagonistic activity against MoT in our laboratory [21]. Among them, the two most potential macrolides, oligomycin B and oligomycin F, previously extracted from the marine *Streptomyces* spp. [3–4] were selected for this study. The specific objectives of this study were to: (i) test the effect of oligomycin B and F on the inhibition of mycelial growth of MoT; (ii) examine their effect on conidiogenesis, conidial germination and subsequent morphological development; (iii) evaluate the suppression of wheat blast disease using detached wheat leaves; and iv) compare the efficacy of these two oligomycins with a local standard fungicide product.

## Materials and methods

### Chemicals

**Fig 1:**
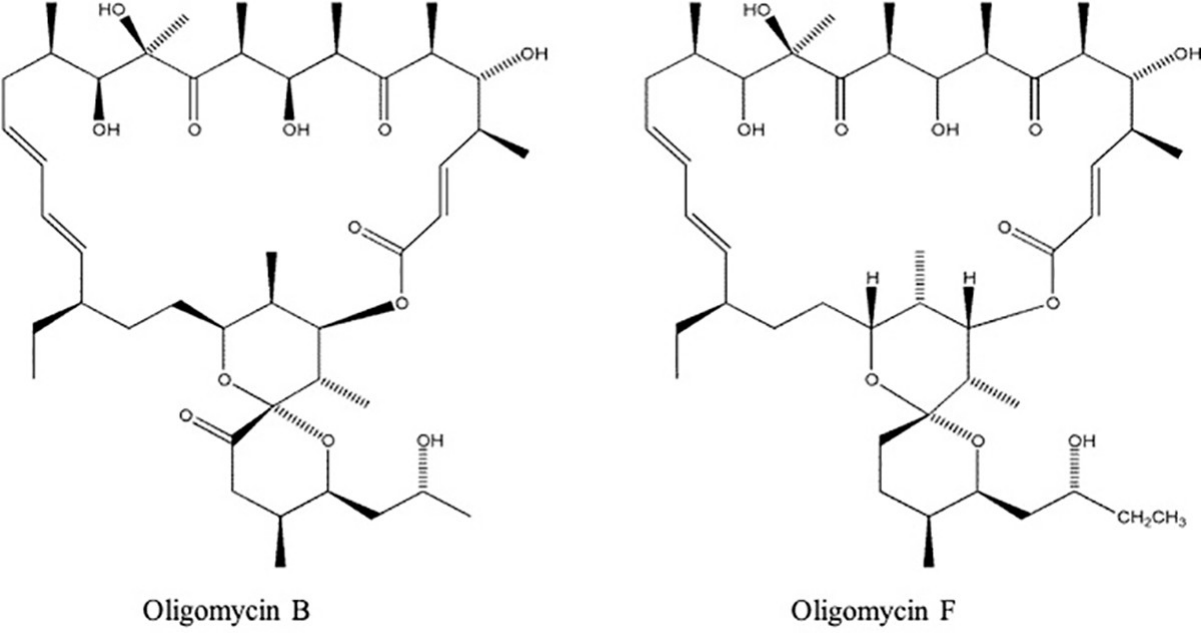
Structures of oligomycin B and F.

Oligomycin B and oligomycin F (Fig 1) were isolated from the marine bacteria, *Streptomyces* sp. strains B8496, B8739 and A171 [3–4]. These pure compounds were generously provided by Dr. Hartmut Laatsch of Georg-August University Goettingen, Germany. The fungicide Nativo® WG 75 (a combination of tebuconazole, 50% and trifloxystrobin, 25%) was purchased from Bayer Crop Science Ltd. Dhaka, Bangladesh.

### Fungal strain, growth media and plant materials

The strain BTJP 4 (5) of MoT was isolated from blast infected spikelets of wheat cv. BARI Gom-24 (Prodip) in Jhenaidah, Bangladesh in 2016. For this research a pure culture from a single spore was preserved at 4°C on dry filter paper [14]. The isolate (BTJP 4) was re-cultured on Potato Dextrose Agar 42 g/L (PDA) at 25°C for 7 days. Ten-day-old fungal cultures grown on PDA were washed in an aseptic environment in a laminar flow hood with 500 ml of deionized distilled water to remove aerial mycelia; then kept at ambient room temperature (25–30°C) for 2–3 days to induce abundant conidia production [14, 29]. Conidia were scraped from the plates with a glass slide after adding 15 ml water into each plate. The conidial and mycelial suspension was filtered through two layers of cheese cloth and adjusted to a concentration of 1 × 10^5^ conidia/ml. Conidial germination was visualized and counted under a compound microscope. Wheat blast susceptible, five-leaf stage seedlings of cultivar BARI Gom-24 (Prodip) was used for the leaf bioassay [30].

### Inhibition of mycelial growth and morphological effects on hyphae

A modified disk diffusion technique [31] was used to determine hyphal growth suppression of MoT isolate BTJP by the oligomycins and the commercial fungicide, Nativo ® WG. A series of concentrations ranging from 0.005 to 2 μg / disk of the oligomycins and the fungicide Nativo ® WG75 were prepared by dissolving required amounts in ethyl acetate and water, respectively. Filter-paper disks (Sigma-Aldrich Co., St. Louis, MO, USA) measuring nine-millimeter diameter were soaked with the test compounds. The treated disks were placed 2 cm from one side of 9 cm dia Petri dishes containing 20 ml PDA. Five-millimeter diameter mycelial plugs from actively growing seven-day-old PDA cultures of MoT were placed on the opposite side of filter paper disk containing test compounds. Petri dishes inoculated with fungal mycelial plugs against fungicide Nativo ® WG75 were used as an industry standard. Filter paper disks treated with ethyl acetate followed by evaporation of ethyl acetate in room temperate served as a negative control. Inhibition of fungal growth was apparent within 10 days of incubation. Plates were incubated at 25°C until the fungal colony fully covered the agar surface of the control plates. There were five replications for each concentration and the experiment was repeated five times. The fungal colony's radial growth was measured in centimeters with a ruler along with two perpendicular lines drawn on each plate's lower side. Data were recorded by measuring the inhibition zone created by test compounds and corresponding mycelial growth. Radial growth inhibition percentage (RGIP) (± standard error) [32] was calculated from mean values as:

$$RGIP\;(\%)=\;\frac{Radial\;growth\;in\;control\;plate-Radial\;growth\;in\;treated\;plate}{Radial\;growth\;of\;control}\times 100$$

Hyphal morphology at the leading edge of the colonies facing the treated and control disks were observed with a Zeiss Primo Star microscope at 40X and 100X (100x was an oil emersion lens). Images of the disk diffusion experiment were captured with a Canon DOS 700D digital camera. Images of the hyphae were captured with a Zeiss Axiocam ERc 5s through the microscope.

### Inhibition of conidiogenesis

Stock solutions of each of the oligomycins were prepared in 10 μl of dimethyl sulfoxide (DMSO). Stock solution was then diluted with distilled water to obtain 5, 10 and 100 μg / ml concentrations. The final concentration of DMSO was never higher than 1% (v / v) in the final solution, which does not affect the hyphal growth or sporulation of MoT. Preparation of 5 ml fungicidal suspension of Nativo®WG75 at 5, 10 and 100 μg/ml concentrations was carried out by mixing the required amount of product in distilled water for using it as a positive control. Mycelium of a 10-day-old Petri dish culture of MoT was washed to reduce nutrients and induce conidiogenesis [14, 29]. Ten mm MoT mycelial agar blocks were treated with 50 μl of each compound and Nativo®WG75 at 5, 10 and 100 μg/ml and put into Nunc multi well plates. The same amount of sterile water was applied on the MoT mycelial block with 1% DMSO serving as a negative control. Treated mycelial agar blocks MoT were incubated at 28°C with >90% RH and 14 h light followed by 10 h of darkness. After 24 hours, conidiogenesis was observed with a Zeiss Primo Star microscope at 40x magnification and images captured with a Zeiss Axiocam ERc 5s. There were five replications per treatment and the experiment was conducted five times.

### Inhibition of conidial germination and morphological changes of germinated conidia

A stock solution of each oligomycin was prepared by dissolving 0.1 μg of the compound in 10 μl dimethyl sulfoxide (DMSO) followed by diluting the concentration. of each compound to 0.1μg/ml by adding distilled water. Nativo®WG75 solution was prepared with distilled water at 0.1 μg/ml to use as a positive control. Conidial germination assays were carried out following the protocol described by Islam and von Tiedemann [33]. For each treatment, a 100 μl solution of 0.1 μg/ml was added directly to 100 μl of 1 × 10^5^ conidia/ml of MoT to make a final volume of 200 μl containing 0.05 μg/ml test compound into a well of a 96-multiwell plate. The solution was mixed immediately with a glass rod and incubated at 25°C. Sterilized water with 1% DMSO served as a control. The multiwell plate was incubated in a moisture chamber at 25 ^0^C for 6 h, 12 h and 24 h in the dark. A total of 100 conidia from each of five replicates were examined under a Zeiss Primo Star at 100x magnification. Percent germination of conidia and developmental differences of the germ tubes and appressoria were evaluated and the images were captured with a Zeiss Axiocam ERc 5s. Each treatment and time course was replicated five times and the experiments repeated five times. The percent conidial germination (± standard error) was calculated from mean values as: CG % = (C–T)/C × 100; Where, CG = conidial germination, C = percentage of germinated conidia in control, and T = percentage of germinated conidia in treated samples.

### Development of wheat blast on detached wheat leaves

Stock solutions of oligomycins B and F were prepared using dimethyl sulfoxide (DMSO). Then preparation of 5, 10 and 100 μg/ml concentrations of each compound was carried out in distilled water where the final concentration of DMSO never exceeded 1%. Nativo®WG75 concentrations were 5, 10 and 100 μg/ml. Sterilized water with 1% DMSO served as a negative control. Wheat leaves were separated from five-leaf stage seedlings and placed in plates lined with moist paper towels. Three 20-μl droplets of the freshly prepared test compounds at concentrations mentioned above were placed on three different spots of each leaf, and left for 15 minutes to dry. Each spot was then inoculated with 1 μl conidial suspension containing 1 × 10^5^ MoT conidia/ml followed by incubating dishes at 28°C under 100% relative humidity in dark for first 30 h, then 2 days in continuous light. The test was performed five times independently with 5 replicate samples. The resulting length of wheat blast lesions MoT were measured from 3 leaves per experiment for each treatment and each concentration of compounds.

### Statistical analysis, experimental design/replications

Experiments were performed using a completely randomized design (CRD) to determine biological activities of the pure oligomycin compounds compared to a standard fungicide. Data were analyzed by one-way ANOVA, and mean values were separated by the posthoc statistic of Tukey's HSD (honest significant difference). All statistical analyses were carried out with SPSS (IBM SPSS statistics 16, Georgia, USA) and Microsoft Office Excel 2010 program package. Mean value ± standard error of 5 replications were used in Tables and Figures.

## Results

### Inhibition of mycelial growth and morphological effects on hyphae

**Fig 2:**
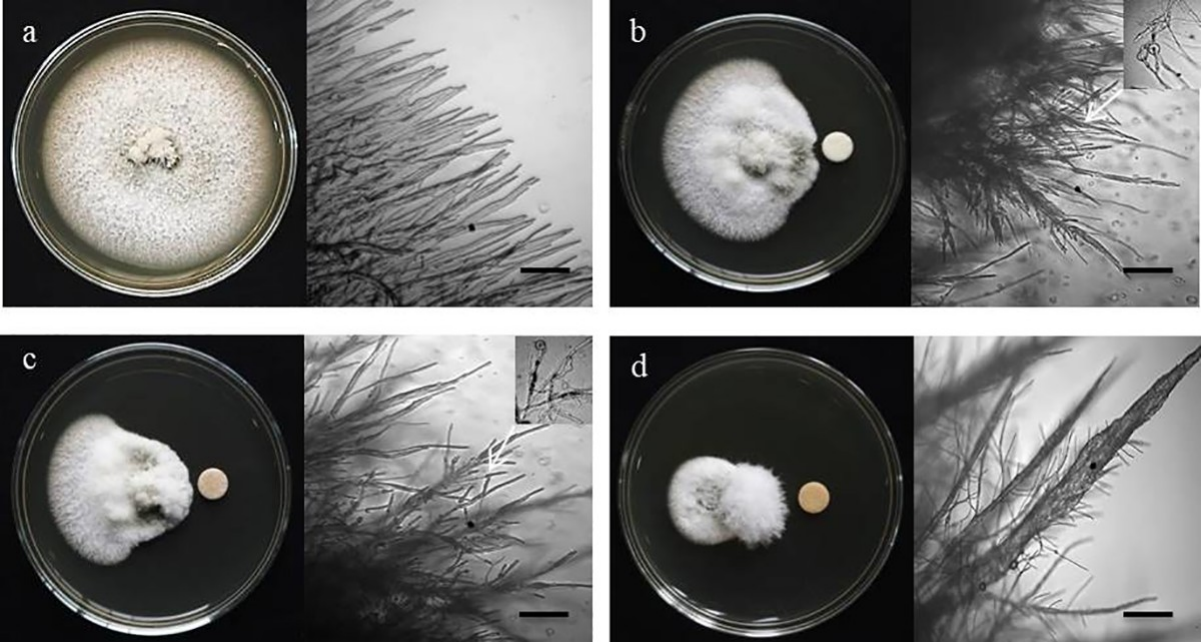
Macroscopic and microscopic images of *in vitro* antifungal activity of oligomycin B, oligomycin F and the commercial fungicide nativo® WG75 against *Magnaporthe oryzae Triticum* at 2 μg/disk; (a) Control, (b) Oligomycin B, (c) Oligomycin F, (d) Nativo® WG75. Bar = 10 μm, 50 μm.

**Fig 3:**
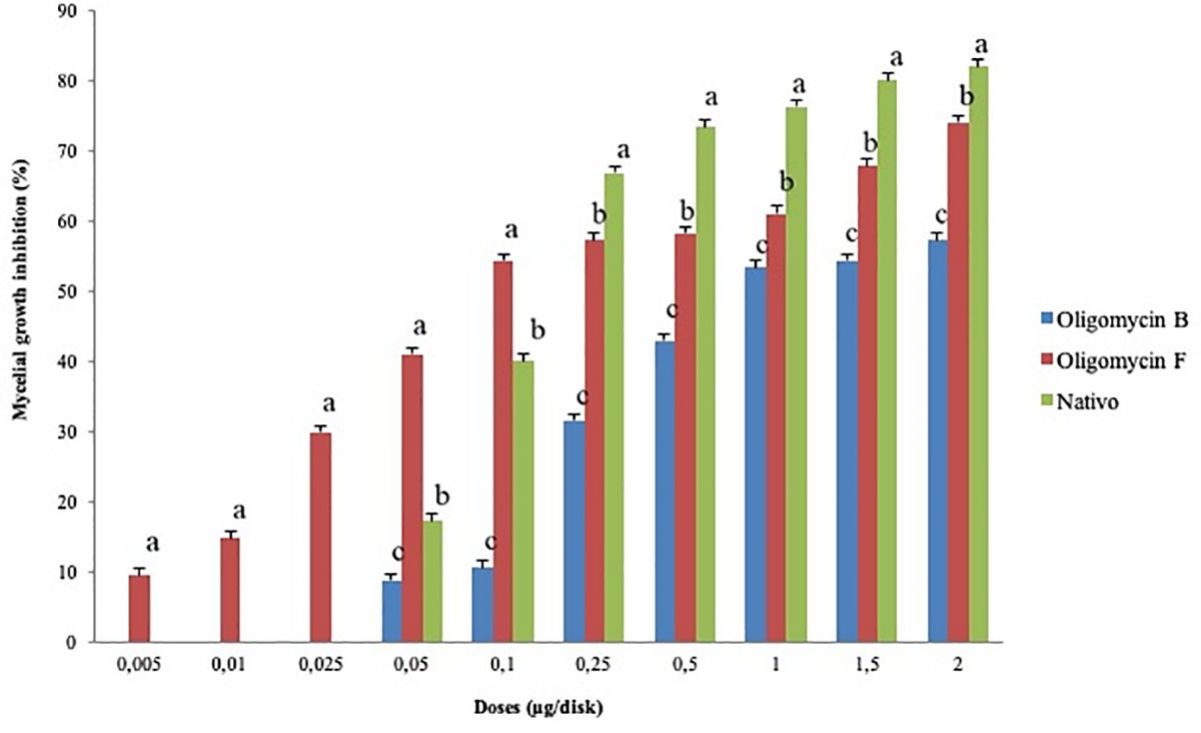
Inhibitory effects of oligomycin B, oligomycin F and the commercial fungicide Nativo® WG75 on hyphal growth of *Magnaporthe oryzae Triticum* in potato dextrose agar. The data are the mean ± standard errors of five replicates for each concentration of the compound tested at a 5% level based on the Tukey HSD (Honest Significance Difference) post-hoc statistic.

Both oligomycins B and F (Fig 1) tested in this study and originally extracted from a *Streptomyces* species showed significant inhibition of MoT hyphal growth on PDA (Fig 2). Between these two compounds, oligomycin F depicted stronger inhibition of hyphal growth of MoT. Mycelial growth inhibition by oligomycins B and F was 57.1 ± 1.3% and 73.9 ± 2.5%, respectively when both compounds were used at 2 μg/disk (Fig 3) The commercial fungicide Nativo® WG 75 had a higher inhibition capacity (81.9 ± 0.9% at 2 μg/disk) than both oligomycin F and B.

Both oligomycins inhibited MoT mycelial growth in a dose dependent manner. Suppressive effects of oligomycin increased with increasing concentrations from 0.005 to 2 μg/disk reaching 74% for oligomycin F (Fig 3). Suppression by oligomycin F was slightly lower than suppression by Nativo® WG 75 but higher than oligomycin B. Neither of the oligomycins showed activity against MoT at concentration lower than 0.005 μg. Oligomycin F showed extensive inhibition of hyphal growth at 2 μg/disk (73.9 ± 2.5%) followed by 1.5 μg/disk (67.6 ± 0.9%) and 1 μg/disk (60.9 ± 2.5%) showing a positive correlation of suppression with an increase in concentration. The percent suppression by oligomycin B was 57.1 ± 1.3%, 54.3 ± 1.3% and 53.3 ± 1.5%, at 2, 1.5 and 1 μg/disk, respectively. The minimum inhibitory concentrations of oligomycin F and oligomycin B were 0.005 and 0.05 μg/disk, respectively. At the minimum inhibitory concentrations, hyphal growth inhibition was 11.4 ± 2.3% and 8.63 ± 1.3%, respectively for oligomycin F and B. The minimal inhibitory concentration of Nativo® WG 75 was 0.05 μg/disk, similar to oligomycin B although fungicide at higher concentration starting from 0.25 μg/disk superseded inhibition percentage at equivalent concentrations of oligomycins. It is interesting to note that at concentrations below 0.25 μg/disk, the inhibition of mycelial growth by oligomycin F was higher than that of the fungicide Nativo® WG 75 and this macrolide displayed inhibitory activity against MoT at about 10-fold lower concentration.

Microscopic observations showed that untreated MoT hyphae had polar, tubular growth with smooth, branched, hyaline, plump, septate, and intact hyphae (Fig 2A). Hyphae treated with oligomycin B and F showed irregular growth and an increase in branch frequency per unit length of the hyphae. The hyphal cell walls were not smooth but showed ridges giving a corrugated appearance and irregular swelling of cells (Fig 2B and 2C). Nativo ® WG75 showed a similar pattern of hyphal growth inhibition. Similar abnormality of MoT also occurred where the hyphae were close to the Nativo ® WG75 disk (Fig 2D). However, morphological changes of MoT by the two oligomycins were slightly different from those observed with the Nativo®WG75 suggesting a possible different mode of action.

### Inhibition of conidiogenesis

**Fig 4:**
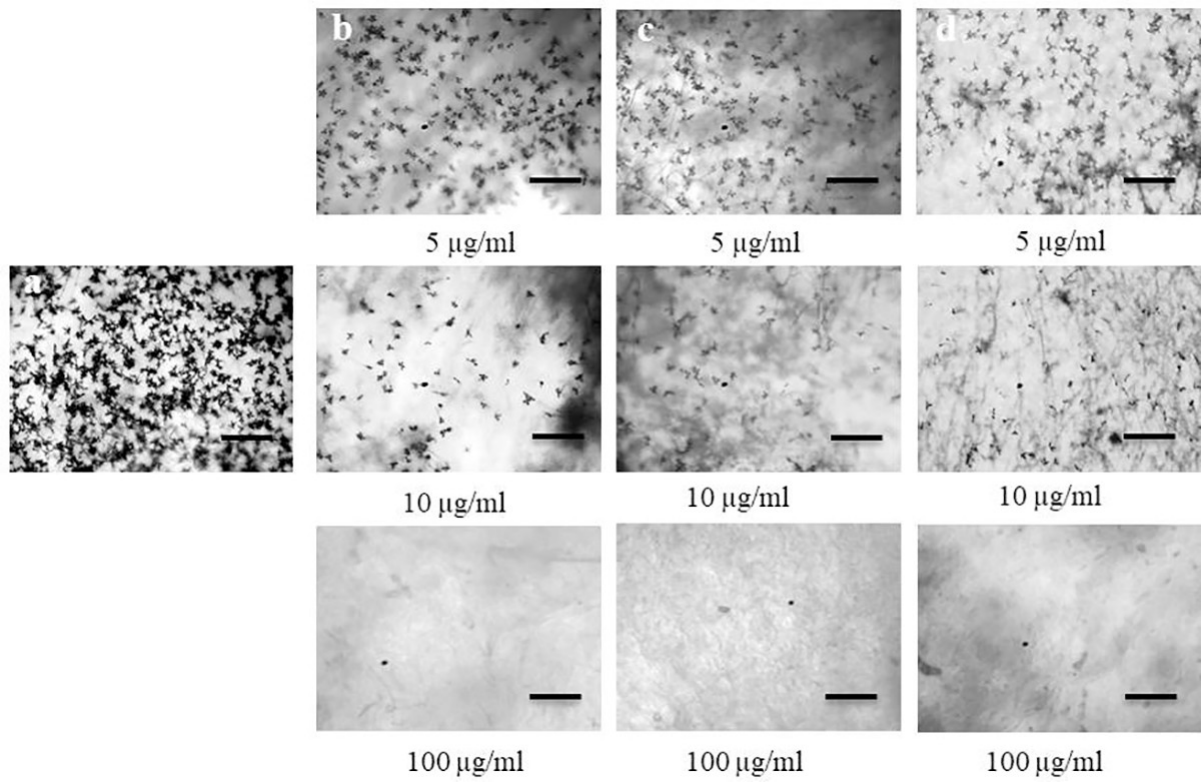
Effects of oligomycin B, oligomycin F and the fungicide Nativo® WG75 on inhibition of conidiogenesis of *Magnaporthe oryzae Triticum* in 96-multiwell plates at 5 μg/ml, 10 μg/ml, 100 μg/ml. (a) Control, (b) Oligomycin B, (c) Oligomycin F, (d) Nativo® WG75. Bar = 50 μm.

Conidial formation by MoT was remarkably decreased by the oligomycins and the fungicide at 5 and 10 μg/ml when compared to the control, and inhibition increased with an increase in concentration from 5, 10 and 100 μg/ml (Fig 4). For all three treatments, no conidia developed at 100 μg/ml. Microscopic examination revealed broken mycelial tips, and a complete lack of conidiophores for all three treatments at 100 μg/ml.

### Inhibition of conidial germination and morphological changes of germinated conidia

Oligomycin B, F and Nativo ® WG75 at 0.05 μg/ml were used to determine the inhibition of conidial germination of MoT in multi-well plates. After 6, 12 and 24 h of incubation, the percent of germinated conidia was recorded (Table 1). After 6 h, all three treatments significantly reduced germination of conidia compared to the control. Germination was 100% in water, and 50.3±0.7% in plates treated with Nativo ® WG75. With oligomycin B and F germination percentages of MoT conidia were 24 ± 0.9% and 53±0.4% at 0.05 μg/ml, respectively.

**Table 1:** Effects of oligomycins and the fungicide Nativo® WG75 on germination of conidia and morphology of germ tubes and appressoria of *Magnaporthe oryzae Triticum* at 0.05 μg/ml *in vitro*.

Compound	Time (h)	Germination of conidia, and morphology of germ tubes and appressorial formation	
		Germinated conidia (% ± SE^a^)	Morphological change/developmental transitions in the treated conidia
Water	0	0 ± 0e	No germination
	6	100 ± 0a	Germination with normal germ tube and normal appressoria
	12	100 ± 0a	Normal mycelial growth
	24	100 ± 0a	Normal mycelial growth
Oligomycin B	0	0 ± 0e	No germination
	6	38.3 ± 0.7d	24 ± 0.9% Short germ tube and 14.3 ± 0.7% conidia lysed
	12	24 ± 0.9d	10.6 ± 0.7% Normal germ tube, 8.4 ± 0.2% short and 5 ± 0.4% Abnormally elongated germ tube
	24	0 ± 0b	No appressoria, no mycelial growth
Oligomycin F	0	0 ± 0e	No germination
	6	59 ± 0.8b	53 ± 0.4% Short germ tube and 6 ± 0.8% conidia lysed
	12	53 ± 0.4b	33.3 ± 0.5% Normal germ tube and 19.7 ± 0.2% abnormal branching at the tips
	24	0 ± 0b	No appressoria, no mycelial growth
Nativo	0	0 ± 0e	No germination
	6	50.3 ± 0.7c	Germinated with a short germ tube
	12	50.3 ± 0.7c	Normal germ tube
	24	0 ± 0b	No appressoria; no mycelial growth

^a^The data presented here are the mean value ± SE of three replicates in each compound. Means within the column followed by the same letter(s) are not significantly different from those assessed by Tukey's HSD (Honest Significance Difference) post-hoc (p ≤ 0.05). Conidia germination percent at different incubation times is not cumulative, rather at different time intervals.

**Fig 5:**
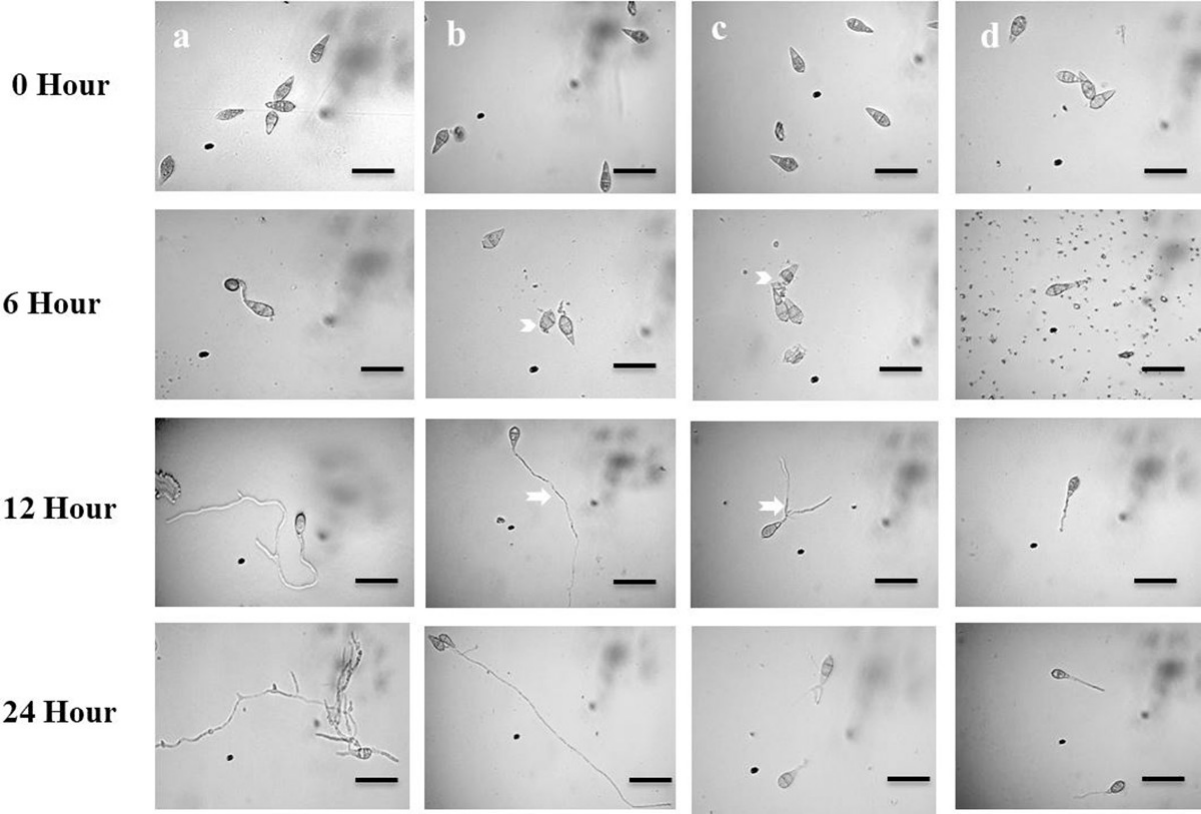
Time-dependent alterations in *Magnaporthe oryzae Triticum* germination of conidia and subsequent morphological changes in the presence of oligomycin B, oligomycin F and the commercial fungicide Nativo® WG75. Dose of oligomycins was 0.05 μg/ml. (a) Control, (b) Oligomycin B, (c) Oligomycin F, (d) Nativo® WG75. Branched germ tube (*arrow*); Elongated germ tube (*arrow*); Lysis of conidia (*arrow head*). Bar = 10 μm.

Conidial germination in water was 100% with normal germ tube development and mycelial growth at all incubation times (6 h, 12 h and 24 h) at 25°C in dark (Table 1, Fig 5A). The two oligomycins had adverse effects not only on conidial germination but also post-germination developmental processes with abnormal transitions from one step to the next at 0.05 μg/ml. Out of 38% of the germinated spores in the oligomycin B treatment, 24 ± 0.9% had short germ tubes and 14.3± 0.7% of the germ tubes lysed within the first 6 h of incubation. After 12 h of incubation in the same treatment, out of 24% germinated spores, 10.6 ± 0.7% were normal, 8.4 ± 0.2% had shorter germ tubes than the control and 5 ± 0.4% had abnormally elongated germ tubes. After 24 h of incubation, no conidia germinated (Table 1, Fig 5B). In the presence of oligomycin F, 53 ± 0.4% of conidia germinated with shorter germ tubes than the control and 6 ± 0.8% conidia lysed after 6 h. Similar developmental abnormalities were found among the germinated conidia after 12 h of incubation with 33.3 ± 0.5% normal and 19.7 ± 0.2% with abnormally branched germ tubes formation, while no germination after 24 h (Table 1, Fig 5C). In the presence of Nativo® WG75, 50.3 ± 0.7% conidia germinated with normal germ tubes after 6h and 12 h, but no appressoria developed. Nativo® WG75 also prevented spore germination after 24 h (Table 1, Fig 5D). It is interesting that the oligomycins produced abnormally long or short or branched germ tubes and lysing of conidia, while the fungicide did not result in these changes.

### Development of wheat blast on detached wheat leaves

**Fig 6:**
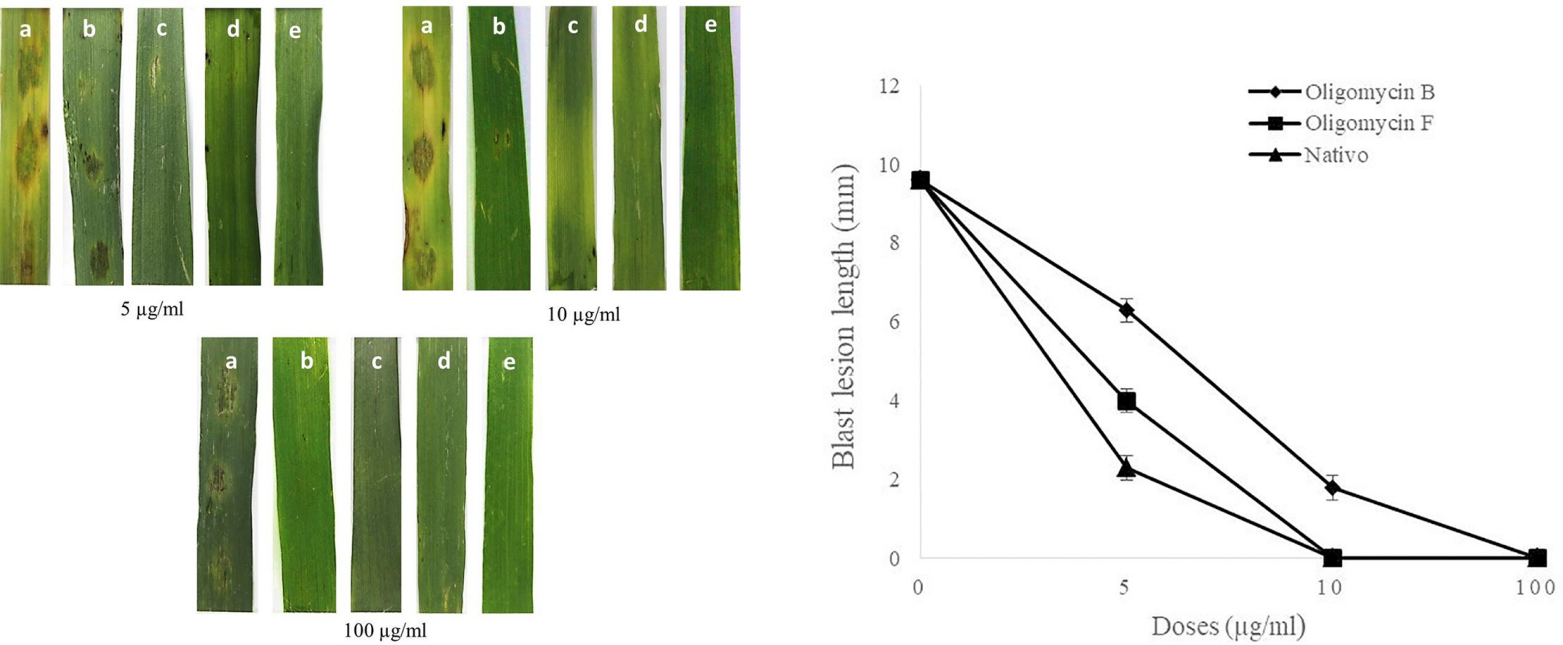
Suppression of wheat blast symptoms with oligomycins at 5 μg/ml, 10 μg/ml and 100 μg/ml on a representative detached wheat leaf of five replicates inoculated with *Magnaporthe oryzae Triticum* (A) blast lesions on treated and untreated wheat leaves (a) Water control+MoT, (b) Oligomycin B+MoT inoculation, (c) Oligomycin F+MoT inoculation, (d) Nativo® WG75+MoT inoculation, (e) Non-inoculated, non-treated leaf; (B) Blast lesion lengths on detached wheat leaves treated with oligomycin B oligomycin F and Nativo ® WG75 fungicide compared with water treatment control. The data are the averages ± standard errors of at least five replicates for each dose of the tested compounds at p ≤ 0.05. Bars represent ± standard error.

Application of the two oligomycins at 5, 10 and 100 μg/ml remarkably inhibited symptoms of wheat blast in detached wheat leaves, inoculated with MoT. The average length of lesions in the wheat leaves treated with oligomycin B were 6.3 ± 0.3 mm and 1.8±0.3 mm at 5μg/ml and 10 μg/ml, respectively (Fig 6A and 6B). With oligomycin F and Nativo®WG75, blast lesion lengths were 4 ± 0.3 mm and 2 ± 0.3 mm at 5 μg/ml, respectively (Fig 6A and 6B). No blast symptoms were visible when leaves were treated with oligomycin F and the fungicide Nativo®WG75 at 10 μg/ml and 100 μg/ml (Fig 6A and 6B). No visible blast lesion occurred when treated with oligomycin B at 100 μg/ml. Leaves treated with water as a negative control developed typical blast lesions with an average length of 9.58 ± 0.2 mm (Fig 6A and 6B). These results show that the fungicide suppressed lesion development more than both oligomycins at 5 μg/ml, the fungicide showed more suppression than oligomycin F at 10 μg/ml, and no lesions developed with any of the three treatments at 100 μg/ml.

## Discussion

In this study, we found that two *Streptomyces* macrolides, oligomycin B and oligomycin F, demonstrated extensive antifungal activities against the devastating wheat blast pathogen of wheat. Of the two oligomycins tested, biological activity of oligomycin F was superior than oligomycin B and in some cases, superior to the commercial fungicide Nativo® WG75. These findings indicate that suppression of conidial germination, appressorial formation and mycelial growth by these macrolides are correlated with wheat blast disease suppression on inoculated leaves. Hyphal growth inhibition, conidial formation and germination of conidia of various fungi including the rice blast fungus (MoO) by a number of natural secondary metabolites from *in vitro* bioassays have been documented by many investigators [3, 11, 34–39]. To the best of our knowledge, this is the first report of suppression of the devastating wheat blast fungus by oligomycin B and F isolated from the *Streptomyces* spp., with the potential to be utilized for managing the disease *in vivo*.

Oligomycins are macrolide antibiotics that block the proton channel (F0 subunit) requisite for the oxidative phosphorylation of ADP to ATP due to inhibition of ATP synthase. [40]. While oligomycins have excellent biological properties, only a few studies have so far focused on the development of plant disease protection products from these macrolides. Interestingly, our result also revealed that the efficacy of oligomycin F in controlling wheat blast fungus was 10-fold stronger than the commercial fungicide Nativo ® WG75 in terms of mycelial growth inhibition.

One of the noteworthy findings from the present study is the induction of swelling on the MoT hyphae by these macrolides (Fig 2B and 2C), which is often considered as a reliable mode of inhibitory action of a compound against the normal growth and development of a fungal pathogen [38, 39]. We tested a range of concentrations of these compounds from 0.005 to 2 μg /disk, and found that swelling in hyphae increased with increasing concentrations of the oligomycins (data not shown). Swelling in various fungal hyphae has been reported earlier by polyoxin B [41], fengycin [37–39] and tensin [42]. Morphological changes like extensive branching and swelling of hyphae of an oomycete pathogen *Aphanomyces cochlioides* by phloroglucinols extracted from *Pseudomonas fluorescence* or xanthobaccin A isolated from *Lysobacter* sp. SB-K88 have been documented [43–46]. According to a report by Kim et al. [11], oligomycin A from *Streptomyces libani*, significantly inhibited mycelial growth of *Magnaporthe grisea*, *Botrytis cinerea*, *Colletotrichum lagenarium*, *Cylindrocarpon destructans*, *Cladosporium cucumerinum and Phytophthora capsici*. So far, this is the first report of swollen-like structures development in hyphae by oligomycins toward the destructive phytopathogen, MoT. Further investigation is needed to understand the detailed modes of action of these macrolides towards suppression of the phytopathogen, MoT.

Conidia are the infecting propagule by which most pathogenic fungi invade plants and the process by which conidia are formed is known as conidiogenesis [35, 47]. The more conidia a fungal pathogen produces the more its potential to destroy a plant, which is very significant in case of an economically important cereal crop like wheat. Inhibition of conidiogenesis and conidial germination reduces the chance of secondary infection. Compounds that are found to inhibit these processes are great candidate for downstream application as plant protection products. A few interesting findings of this study showed that both oligomycin B and F not only strongly suppressed conidiogenesis (Fig 4), but also inhibited germination of conidia and further morphological advancement of the germ tube towards hyphal growth (Table 1, Fig 5). Results from the bioassay showed that wheat leaves prophylactically treated with these macrolides at 5, 10 and 100 μg/ml had restricted fungal growth and limited disease development. Other novel and interacting phenomena observed in this study included lysis of conidia, irregular branching of the tip of germ tube, and abnormally elongated hypha-like germ tubes (Fig 5B and 5C). Similar phenomenon was observed by Dame et al., [4] who reported that oligomycin A, B and F from a marine *Streptomyces* could induce lysis of zoospores of the plant pathogen *Plasmopara viticola*, which causes grapevine downy mildew. Homma et al. [48] reported that lecithin induced abnormal branching at the conidial germ tube tips and inhibited appressoria formation of rice blast fungus. Islam and Fukushi [46] reported that cystospores of *A*. *cochlioides* produced in the presence of diaacetylphloroglucinol (DAPG) subsequently germinated with hyperbranched germ tubes. Modes of action and mechanism of inhibition of conidiogenesis, germination and formation of appressoria of the MoT conidia by oligomycins has not been previously reported.

Oligomycins are macrolide antibiotics that impede ATP production by influencing oxidative phosphorylation in mitochondria [49]. The oligomycin comprises a 26-membered α, β-unsaturated lactone with a conjugated diene fused to a bicyclic spiroketal ring system. Their mode of action includes the decoupling of mitochondrial ATPase F0 and F1 factors responsible for promoting the transfer of proton via the inner mitochondrial membrane [50]. The enzymatic complex F0F1 ATP synthase may be considered as a target for antifungal and anti-tumor or anti-infection therapy [51]. Oligomycins display a number of important biological activities including mitochondrial ATPase inhibition, strong antifungal, anti-actinobacterial and anti-tumor effects that have been reported [9, 1, 51]. These natural products are among the strongest selective agents in the cell line; they interrupt P-glycoprotein activity and induce apoptosis in doxorubicin-resistant HepG2 cells [52]. Oligomycins have a variety of isomers called oligomycin A through G. These are particularly relevant to the disruption of mitochondrial metabolism [10]. Moreover, elucidation of structure has provided new horizons for developing new ATP synthase-directed agents with possible therapeutic effects [53]. The first reports of chemical modification of oligomycin A have already been documented by Lysenkova et al. [54]. New compounds also showed efficacy against *Candida albicans*, *Aspergillus niger* and *Cryptococcus humicolus*, with other biological properties similar to those of oligomycin A, but with less cytotoxic effects. During germination, conidia might need a constant energy (ATP) supply from the internal energy reserve of the cells [55]. Therefore, a plausible explanation for the suppression of hyphal growth and conidia germination of MoT demonstrated in this study is likely to be associated with ATP synthesis inhibition in mitochondria due to the effects of oligomycins. More studies are required to determine the precise structure-activity relationships of these oligomycins, which may make it possible to synthesize a more active oligomycin as an effective agrochemical against MoT.

A hallmark finding of this study is that application of both macrolides significantly inhibited blast disease development in detached leaves of wheat (Fig 6). In this study, wheat leaves treated with oligomycin B and F had shorter lesion lengths than the untreated control (Fig 6). Many of the lesions in treated leaves were small brown in color with pinhead-sized specks (scale 1) to small, roundish to slightly elongated infecting <10% of wheat leaf area (scale 5). In contrast, water treated control leaves had typical blast lesions infecting more than 75% wheat leaf area (scale 9) corresponding to scale 9 of blast disease assessment scale provided by the IRRI Standard Evaluation System [56]. However, no blast lesions were visible on the leaves treated with the oligomycin compounds and Nativo ® WG75 at the highest concentration (Fig 6). Nativo ® WG75 is a systemic wide-spectrum commercial fungicide that we used as a positive control. Interestingly, the antifungal effect of oligomycins on the inhibition of MoT fungus was found equivalent or stronger than that of the fungicide. Tebuconazole and trifloxystrobin are two main active ingredients of Nativo ® WG75. Tebuconazole is known as a demethylase inhibitor (DMI), which is a systemic triazole fungicide. Demethylase inhibitors inhibit ergosterol biosynthesis, which is a major component of the plasma membrane of certain fungi essential for growth and further development of the fungus [57]. Trifloxystrobin is a strobilurin fungicide that interferes with the respiration of plant pathogenic fungi by preventing energy production in mitochondria, thereby inhibiting germination of fungal conidia [58]. The mechanisms of disease suppression by the oligomycins are likely different compared with the mechanisms of Nativo ® WG75 although similar disease suppression effect has been obtained. Further study is needed to elucidate the underlying mechanism of wheat blast disease suppression by the oligomycin B and F. Furthermore, a field trial of the oligomycins in controlling wheat head infection is needed before considering them as effective fungicides against the wheat blast.

Despite their significant potency as antifungal compounds, little information is available on the effectiveness of oligomycins as agricultural fungicides. The shorter residual effect of oligomycin can be important in the reduction of deleterious effects on humans and the environment, considering that sufficient efficacy in the management of plant diseases is sustained [11]. Oligomycin A was found the most active anti-filamentous fungal analogue among antibiotics in the oligomycin family [1, 59, 60]. As oligomycin F is the immunosuppressive homolog of oligomycin A [3], and oligomycin B is a stable natural product [61], these macrolides have the potential to be the leading compounds for the production of agrochemicals against the cereal killer MoT.

Frequent application of commercial fungicides with site-specific modes of action, like strobilurins (QoI, quinone outside inhibitors) and triazoles, has resulted in the widespread occurrence of resistant mutant species in MoT [27, 28]. Resistance development in fungal population against fungicides prompted search for new, effective antifungal agents with alternate mode of action to protect wheat plants against this phytopathogenic fungus. Results from this study pertaining to the inhibitory ability of these macrolides should motivate agrochemical companies to consider these as candidates for commercial products with novel modes of action against the wheat blast fungus.

## Conclusion

Our findings show that oligomycin B and F from *Streptomyces* spp., suppress hyphal growth and asexual development of MoT, and inhibited wheat blast development on detached leaves of wheat. Field assessment of these macrolides is required to evaluate these metabolites as effective fungicides against wheat blast. Further research is also required to understand the mode of action and the structure-activity relations among oligomycins A-G against the devastating wheat killer, *M*. *oryzae Triticum*.

## References

[1] Masamune S, Sehgal JM, van Tamelen EE, Strong FM, Peterson WH. Separation and preliminary characterization of oligomycins A, B and C. J Am Chem Soc. 1958; 80: 6092–95. 10.1021/ja01555a049

[2] Yamazaki M, Yamashita T, Harada T, Nishikiori T, Saito S, Shimada N, Fujii, A. 44-homooligomycins A and B, new antitumor antibiotics from *Streptomyces bottropensis* producing organism, fermentation, isolation, structure elucidation and biological properties. J Antibiot. 1992; 45: 171–79. 10.7164/antibiotics.45.171 1556008

[3] Laatsch H, Kellner M, Wolf G, Lee YS, Hansske F, Konetschny-Rapp S, et al Oligomycin F, a new immunosuppressive homologue of oligomycin A. J Antibiot. 1993; 46: 1334–41. 10.7164/antibiotics.46.1334 8226311

[4] Dame ZT, Islam MT, Helmke E, von Tiedemann A, Laatsch H. Oligomycins and pamamycin homologs impair motility and induce lysis of zoospores of the grapevine downy mildew pathogen, *Plasmopara viticola*. FEMS Microbiol Lett. 2016; 363(16) fnw167: 1–6. 10.1093/femsle/fnw167 27354061

[5] Newitt JT, Prudence SMM, Hutchings MI, Worsley SF. Biocontrol of cereal crop diseases using *Streptomycetes*. Pathogens. 2019; 8: 78. 3390/pathogens8020078. 10.3390/pathogens8020078 PMC6630304 31200493

[6] Ōmura S. Microbial metabolites: 45 years of wandering, wondering and discovering. Tetrahedron. 2011; 67 (35): 6420–59. 10.1016/j.tet.2011.03.117

[7] Enomoto Y, Shiomi K, Matsumoto A, Takahashi Y, Iwai Y, Harder A, et al Isolation of a new antibiotic oligomycin G produced by Streptomyces sp. WK-6150. J Antibiot. 2001, 54: 308–13. 10.7164/antibiotics.54.308 11372788

[8] Salomon AR, Voehringer DW, Herzenberg LA, Khosla C. Apoptolidin, a selective cytotoxic agent, is an inhibitor of F0F1-ATPase. Chem Biol. 2001; 8: 71–80. 10.1016/s1074-5521(00)00057-0 11182320

[9] Kobayashi K, Nishino C, Ohya J, Sato S, Mikawa T, Shiobara Y, et al Oligomycin E, a new antitumor antibiotic produced by *Streptomyces* sp. MCI-2225. J Antibiot. 1987; 40, 1053–57. 10.7164/antibiotics.40.1053 3624067

[10] Shin YK, Yoo BC, Chang HJ, Jeon E, Hong SH, Jung MS, et al Down-regulation of mitochondrial F1F0-ATP synthase in human colon cancer cells with induced 5-fluorouracil resistance. Cancer Res. 2005; 65(8): 3162–70. 10.1158/0008-5472.CAN-04-3300 15833846

[11] Kim BS, Moon SS, Hwang BK. Isolation, identification, and antifungal activity of a macrolide antibiotic, oligomycin A, produced by *Streptomyces libani*. Can J Bot. 1999; 77: 850–58. 10.1139/b99-044

[12] Igarashi S, Utiamada CM, Igarashi LC, Kazuma AH, Lopes RS. Pyriculariaemtrigo. 1. Ocorrência de Pyricularia sp. no estado do Paraná. Fitopatol Bras. 1986; 11: 351–52.

[13] Kohli MM, Mehta YR, Guzman E, Viedma L, Cubilla LE. *Pyricularia* blast-a threat to wheat cultivation. Czech J Genet Plant Breed. 2011; 47: 130–34.

[14] Islam MT, Croll D, Gladieux P, Soanes DM, Persoons A, Bhattacharjee P, et al, Emergence of wheat blast in Bangladesh was caused by a South American lineage of *Magnaporthe oryzae*. BMC Biol. 2016; 14, 84 10.1186/s12915-016-0309-7 27716181 PMC5047043

[15] Islam MT, Kim KH, Choi J. Wheat blast in Bangladesh: the current situation and future impacts. Plant Pathol J. 2019; 35(1): 1–10. 10.5423/PPJ.RW.08.2018.0168 30828274 PMC6385656

[16] Tufan HA, McGrann GRD, Magusin A, Morel JB, Miche L, Boyd LA. Wheat blast: histopathology and transcriptome reprogramming in response to adapted and non-adapted *Magnaporthe* isolates. New Phytol. 2009; 184(2): 473–84. 10.1111/j.1469-8137.2009.02970.x 19645735

[17] Ceresini PC, Castroagudín VL, Rodriguez FA, Rios JA, Aucique-Pérez CE, Moreira SI, et al Wheat blast: from its origin in South America to its emergence as a global threat. Mol Plant Pathol. 2019; 20(2): 155–72. 10.1111/mpp.12747 30187616 PMC6637873

[18] Wilson RA, Talbot NJ. Under pressure: investigating the biology of plant infection by *Magnaporthe oryzae*. Nat Rev Microbiol. 2009; 7(3): 185–95. 10.1038/nrmicro2032 19219052

[19] Inoue K, Suzuki T, Ikeda K, Jiang S, Hosogi N, Hyon GS, et al Extracellular matrix of *Magnaporthe oryzae* may have a role in host adhesion during fungal penetration and is digested by matrix metalloproteinases. J Gen Plant Pathol. 2007; 73: 388–98. 10.1007/s10327-007-0048-2

[20] Urashima AS, Hashimoto Y, Le Don D, Kusaba M, Tosa Y, Nakayashiki, et al Molecular analysis of the wheat blast population in Brazil with a homolog of retrotransposon MGR583. Jpn J Phytopathol. 1999; 65: 429–36. 10.3186/jjphytopath.65.429

[21] Chakraborty M, Mahmud NU, Gupta DR, Tareq FS, Shin HJ, Islam T. Inhibitory effects of linear lipopeptides from a marine *Bacillus subtilis* on the wheat blast fungus *Magnaporthe oryzae Triticum*. Front Microbiol. 2020; 11(665): 1–14. 10.3389/fmicb.2020.00665 32425899 PMC7203576

[22] Callaway E. Devastating wheat fungus appears in Asia for first time. Nature. 2016; 532: 421–422. 10.1038/532421a 27121815

[23] CIMMYT. Wheat Blast Disease: A Deadly and Baffling Fungal Foe. International Maize and Wheat Improvement Center; Texcoco, Mexico. 2016.

[24] Mundi. Agricultural production, supply, and distribution: wheat production by country in 1000 MT. 2016.

[25] Yoon MY, Cha B, Kim JC. Recent trends in studies on botanical fungicides in agriculture. Plant Pathol J. 2013; 29(1): 1–9. 10.5423/PPJ.RW.05.2012.0072 25288923 PMC4174793

[26] Stefani A, Felício JD, de Andréa MM. Comparative assessment of the effect of synthetic and natural fungicides on soil respiration. Sensors. 2012; 12(3): 3243–3252. 10.3390/s120303243 22737005 PMC3376576

[27] Castroagudín VL, Ceresini PC, De Oliveira SC, Reges JTA, Maciel JLN, Bonato ALV, et al Resistance to QoI fungicides is widespread in Brazilian populations of the wheat blast pathogen *Magnaporthe oryzae*. Phytopathology. 2015; 105: 284–94. 10.1094/PHYTO-06-14-0184-R 25226525

[28] Dorigan AF, Carvalho GD, Poloni NM, Negrisoli MM, Maciel JLN, Ceresini PC. Resistance to triazole fungicides in *Pyricularia* species associated with invasive plants from wheat fields in Brazil. Acta Sci Agron. 2019; 41(1): 39332 10.4025/actasciagron.v41i1.39332

[29] Urashima AS, Igarashi S, Kato H. Host range, mating type, and fertility of *Pyricularia grisea* from wheat in Brazil. Plant Dis. 1993; 77(12): 1211–16. 10.1094/PD-77-1211

[30] He Y, Zhu M, Huang J, Hsiang T, Zheng L. Biocontrol potential of a *Bacillus subtilis* strain BJ-1 against the rice blast fungus *Magnaporthe oryzae*. Can J Plant Pathol. 2019; 41(1): 47–59. 10.1080/07060661.2018.1564792

[31] Bauer AW, Kirby WM, Sherris JC, Turck M. Antibiotic susceptibility testing by a standardized single disk method. Am J Clin Pathol. 1966; 45(4): 493–96. 10.1093/ajcp/45.4_ts.493 5325707

[32] Riungu GM, Muthorni JW, Narla RD, Wagacha JM, Gathumbi JK. Management of *Fusarium* head blight of wheat and deoxynivalenol accumulation using antagonistic microorganisms. Plant Pathol J. 2008; 7: 13–9. 10.3923/ppj.200

[33] Islam MT, von Tiedemann A. 2,4-Diacetylphloroglucinol suppresses zoosporogenesis and impairs motility of Peronosporomycete zoospores. World J Microb Biotechnol. 2011; 27(9): 2071–79. 10.1007/s11274-011-0669-7 22448105 PMC3291839

[34] Romero D, de Vicente A, Olmos JL, Davila JC, Perez-Garcia A. Effect of lipopeptides of antagonistic strains of *Bacillus subtilis* on the morphology and ultrastructure of the cucurbit fungal pathogen *Podosphaera fusca*. J Appl Microbiol. 2007; 103: 969–76. 10.1111/j.1365-2672.2007.03323.x 17897200

[35] Kopecká M, Ilkovics L, Ramíková V, Yamaguchi M. Effect of cytoskeleton inhibitors on conidiogenesis and capsule in the long neck yeast *Fellomyces* examined by scanning electron microscopy. Chemo. 2010; 56(3): 197–202. 10.1159/000316330 20551635

[36] Tang QY, Bie XM, Lu ZX, Lv FX, Tao Y, Qu XX. Effects of fengycin from *Bacillus subtilis* fmbJ on apoptosis and necrosis in *Rhizopus stolonifer*. J Microbiol. 2014; 2: 675–680. 10.1007/s12275-014-3605-3 25098563

[37] Gond SK, Bergen MS, Torres MS, White JF Jr. Endophytic *Bacillus* spp. produce antifungal lipopeptides and induce host defence gene expression in maize. Microbiol Res. 2015; 172: 79–87. 10.1016/j.micres.2014.11.004 25497916

[38] Liao JH, Chen PY, Yang YL, Kan SC, Hsieh FC, Liu YC. Clarification of the antagonistic effect of the lipopeptides produced by *Bacillus amyloliquefaciens* BPD1 against *Pyricularia oryzae* via In Situ MALDI-TOF IMS Analysis. Molecules. 2016; 21(12): 1670 10.3390/molecules21121670 PMC6273258 27918491

[39] Zhang L, Sun C. Fengycins, cyclic lipopeptides from marine *Bacillus subtilis* strains, kill the plant-pathogenic fungus *Magnaporthe grisea* by inducing reactive oxygen species production and chromatin condensation. Appl Environ Microbiol. 2018; 84: e00445–18. 10.1128/AEM.00445-18 29980550 PMC6122000

[40] Jastroch M, Divakaruni AS, Mookerjee S, Treberg JR, Brand MD. Mitochondrial proton and electron leaks. Essays Biochem. 2010; 47(1): 53–67. 10.1042/bse0470053 20533900 PMC3122475

[41] Isono K, Nagatsu J, Kawashima Y, Suzuki S. Studies on polyoxins, antifungal antibiotics. Agric Biol Chem. 1965; 29(9): 848–54. 10.1080/00021369.1965.10858475

[42] Nielsen TH, Thrane C, Christophersen C, Anthoni U, Sørensen J. Structure, production characteristics and fungal antagonism of tensin–a new antifungal cyclic lipopeptide from *Pseudomonas fluorescens* strain 96.578. J Appl Microbiol. 2000; 89: 992–1001. 10.1046/j.1365-2672.2000.01201.x 11123472

[43] Islam MT, Hashidoko Y, Deora A, Ito T, Tahara S. Suppression of damping-off disease in host plants by the rhizoplane bacterium *Lysobacter* sp. strain SB-K88 is linked to plant colonization and antibiosis against soilborne Peronosporomycetes. Appl Environ Microbiol. 2005; 71: 3786–3796. 10.1128/AEM.71.7.3786-3796.2005 16000790 PMC1169021

[44] Islam MT. Disruption of ultrastructure and cytoskeletal network is involved with biocontrol of damping-off pathogen *Aphanomyces cochlioides* by *Lysobacter* sp. strain SB-K88. Biol Control. 2008; 46: 312–21. 10.1016/j.biocontrol.2008.02.006

[45] Islam MT. Mode of antagonism of a biocontrol bacterium *Lysobacter* sp. SB-K88 toward a damping-off pathogen *Aphanomyces cochlioides*. World J Microb Biotechnol. 2010; 26: 629–37. 10.1007/s11274-009-0216-y

[46] Islam MT, Fukushi Y. Growth inhibition and excessive branching in *Aphanomyces cochlioides* induced by 2,4-diacetylphloroglucinol is linked to disruption of filamentous actin cytoskeleton in the hyphae. World J Microb Biotechnol. 2010; 26: 1163–70. 10.1007/s11274-009-0284-z 24026919

[47] Ohara T. *REN1* is required for development of microconidia and macroconidia, but not of chlamydospores, in the plant pathogenic fungus *Fusarium oxysporum*. Genet. 2004; 166(1): 113–24. 10.1534/genetics.166.1.113 15020411 PMC1470687

[48] Homma Y, Takahashi H, Arimoto Y. Studies on the mode of action of soybean lecithin. Jpn J Phytopathol. 1992; 58(4): 514–21. 10.3186/jjphytopath.58.514

[49] Smith RM, Peterson WH, McCoy E. Oligomycin, a new antifungal antibiotic. Antibiot Chemother. 1954; 4: 962–70. 24543225

[50] Pagliarani A, Sesci S, Ventrella V. Modifiers of the oligomycin sensitivity of the mitochondrial F1F0-ATPase. Mitochondrion. 2013; 13: 312–19. 10.1016/j.mito.2013.04.005 23597783

[51] Alekseeva MG, Elizarov SM, Bekker OB, Lubimova IK, Danilenko VN. F0F1 ATP synthase of streptomycetes: modulation of activity and oligomycin resistance by protein Ser/Thr kinases. Biochem Moscow Suppl Ser. A 3, 16–23 (2009). 10.1134/S1990747809010036

[52] Li YC, Fung KP, Kwok TT, Lee CY, Suen YK, Kong SK. Mitochondria-targeting drug oligomycin blocked P-glycoprotein activity and triggered apoptosis in doxorubicin-resistant HepG2 Cells. Chemotherapy 2004; 50: 55–62. 10.1159/000077803 15211078

[53] Neupane P, Bhuju S, Thapa N, Bhattarai HK. ATP synthase: structure, function and inhibition. Biomol Concepts. 2019; 10(1): 1–10. 10.1515/bmc-2019-0001 30888962

[54] Lysenkova LN, Turchin KF, Danilenko VN, Korolev AM, Preobrazhenskaya MN. The first examples of chemical modification of oligomycin A. J Antibiot. 2009; 63(1): 17–22. 10.1038/ja.2009.112 19911026

[55] Bimpong CE. Changes in metabolic reserves and enzyme activities during zoospore motility and cyst germination in *Phytophthora palmivora*. Can J Bot. 1975; 53: 1411–16. 10.1139/b75-170

[56] IRRI. Standard Evaluation System for Rice. 4th Edn., International Rice Research Institute (IRRI), Los Banos, Philippines, 1996; Pages: 52.

[57] Pring RJ. Effects of triadimefon on the ultrastructure of rust fungi infecting leaves of wheat and broad bean (*Vicia faba*). Pestic Biochem Phys. 1984; 21(1): 127–37.

[58] Sauter H, Steglich W, Anke T. Strobilurins: evolution of a new class of active substances. *Angew*. Chem Int Ed Engl. 1999; 38: 1328–49. 10.1002/(SICI)1521-3773(19990517)38:10&lt;1328::AID-ANIE1328&gt;3.0.CO;2-1 29711574

[59] Marty EW, McCoy E. The chromatographic separation and biological properties of the oligomycins. Antibiot Chemother. 1959; 9: 286–93. 24545211

[60] Sakagami Y, Ueda A, Yamabayashi S, Tsurumaki Y, Kumon S. A new antibiotic, hondamycin. I. Isolation and characterization. J Antibiot. 1969; 22: 521–27. 10.7164/antibiotics.22.521 5364428

[61] Lardy HA, Witonsky P, Johnson D. Antibiotics as tools for metabolic studies. IV. Comparative effectiveness of oligomycins A, B, C, and rutamycin as inhibitors of phosphoryl transfer reactions in mitochondria. Biochem. 1965; 4: 552–54. 14311628 10.1021/bi00879a027

