# Supplementary material for: Oligomycins inhibit Magnaporthe oryzae Triticum and suppress wheat blast disease
Source: PLoS One. 2020 Aug 17;15(8):e0233665. doi: 10.1371/journal.pone.0233665 (PMC7430738; doi:10.1371/journal.pone.0233665)
Supplement: Attachment — Submitted filename: Manuscript_Oligomycin-Clean Version.docx [file pone.0233665.s001.docx]

**Oligomycins Inhibit *Magnaporthe oryzae triticum* and suppress wheat blast disease**

**Moutoshi Chakraborty^1¶^, Nur Uddin Mahmud^1¶^, Abu Naim Md. Muzahid^1^, S. M. Fajle Rabby^1^, Tofazzal Islam^1^***

^1^Institute of Biotechnology and Genetic Engineering, Bangabandhu Sheikh Mujibur Rahman Agricultural University, Gazipur 1706, Bangladesh

**^¶^**These authors contributed equally to this work.

*Corresponding author:

E-mail: [tofazzalislam@yahoo.com](mailto:tofazzalislam@yahoo.com)

**Abstract**

Oligomycins are macrolide antibiotics, produced by *Streptomyces* spp. that show antagonistic effects against several microorganisms such as bacteria, fungi, nematodes and the oomycete *Plasmopara viticola*. Conidiogenesis, germination of conidia and formation of appressoria are determining factors pertaining to pathogenicity and successful diseases cycle of filamentous fungal phytopathogen.. The goal of this research was to evaluate the in vitro suppressive effects of two oligomycins, oligomycin B and F along with a commercial fungicide Nativo® 75WG on hyphal growth, conidiogenesis, conidia germination, and appressoria formation of the wheat blast fungus, *Magnaporthe oryzae Triticum* (MoT) pathotype. We also determined the efficacy of these two oligomycins and fungicide product in vivo in suppressing wheat blast as detached leaf assay. Both oligomycins suppressed the growth of *MoT* mycelium in a dose dependent manner. Between the two natural products, oligomycin F provided higher inhibition of *MoT* hyphal growth compared to oligomycin B with minimum inhibitory concentrations (MICs) of 0.005 and 0.05 µg/disk, respectively. The application of the compounds completely halted conidial formation of the *MoT* mycelium in agar medium. Further bioassay showed that these compounds significantly inhibited *MoT* conidia germination and induced lysis. The compounds also caused abnormal germ tube and suppressed appressoria formation of germinated spores. Interestingly, the application of these macrolides significantly inhibited wheat blast on detached leaves of wheat. This is the first report on the inhibition of mycelial growth, process of conidia formation, germination of conidia, deleterious morphological changes in germinated conidia, and suppression of blast disease of wheat by oligomycins from *Streptomyces* spp. Further study is needed to unravel the precise mode of action of these natural compounds and consider them as biopesticides for controlling wheat blast

**Introduction**

Oligomycins are macrolide antibiotics, produced by some strains of *Streptomyces* species. They have broad-spectrum biological activities against organisms like fungi, bacteria, nematodes and the oomycete *Plasmopara viticola.* [1-4]. *Streptomyces* species are common soil-dwelling bacteria that have been broadly used as bio-control agents [5]. *Streptomyces* species produce a number of bioactive compounds possessing antifungal, antiviral, antibacterial, anticancer, nematicidal, and antioxidant properties [5, 6]. Several previous studies showed that the effectiveness of some strains of *Streptomyces* in biological control of phytopathogens largely depends on the production of oligomycins [7]. The oligomycins are mitochondrial F1F0 ATP synthase inhibitors that cause apoptosis in a number of cell types [8]. The oligomycin complex, which was first documented in 1954 in a strain of a soil bacterium, *Streptomyces diastatochromogenes* was highly inhibitory against fungi [1]. Antifungal, antitumor, insecticidal, immunosuppressive and nematocidal properties of oligomycins have also been reported [1-3, 7, 9]. The oligomycins contain analog isomers A through G that are highly selective for disrupting mitochondrial metabolism [3, 4, 8, 10]. Although biological activities of oligomycins on fungi and see comment line 14. have been reported, very little is known about the effect of these natural products on a notorious wheat blast fungus *Magnaporthe oryzae triticum* (*Mot*). The bioactivities of oligomycins against different classes of fungal species indicates that their targets may involve a variety of cellular processes, such as inhibition of mycelial growth of *Cladosporium cucumerinum, Magnaporthe grisea, Colletotrichum lagenarium, Botrytis cinerea, Cylindrocarpon destructans, Fusarium culmorum, Erysiphe graminis* and *Phytophthora capsici* [3, 11], lysis and motility inhibition of *P. viticola,* and *Aphanomyces cochlioides* zoospores. ty [4].

The wheat blast fungus *MoT* is one of the most destructive pathogens of wheat [12-15]. The three-celled, hyaline and pyriform fungal conidium attaches to the host surface by secreted adhesive [14, 16, 17]. The attached conidium germinates to form a hyphal germ tube, an aspersorium and a penetration peg to penetrate the epidermis of the host and complete the infection process [16, 18]. The invasion of plant tissue is achieved by bulbous hyphae invaginating the host plasma membrane and penetrating the epidermal cells [16-18]. The fungus can attack wheat plants at any stage of development and infects including leaves, nodes, stems, and spikelets [15, 17, 19]. Mycelium can survive in the embryo, endosperm, and kernal tissues of wheat seed. Wheat blast mainly affects wheat heads; it bleaches the infected heads, resulting in deformed seed or no seed production [14]. The badly affected wheat heads can die, leading to a drastic reduction in grain yield. Bleaching of the spikelets or the entire head at an early stage is the most common recognizable symptom of the disease [12, 14, 15] Infected seeds and airborne conidia usually disseminate the disease, and the fungus may survive in infected seeds and crop residues [20]. Therefore, pyriform conidia developed from conidiophores and conidia germination with appressorial development at the germ tube tips are essential steps of the disease cycle of *MoT* [16]. Disruption of any of these asexual life stages reduces the chance of pathogenesis and development of an epidemic [21]. Finding natural bioactive compounds capable of inhibiting any of these asexual life stages is considered the first step in the development of a new fungicide for controlling *MoT*.

Wheat blast was first found in Brazil in 1985, and subsequently spread to neighboring Bolivia and Paraguay [12, 19]. The cultivation of wheat (*Triticum aestivum*) has increased in Bangladesh in recent years making it the 2^nd^ largest food source after rice. A sudden outbreak of wheat blast disease occurred in Bangladesh in 2016, which was the first incident of wheat blast outside of South America [14, 22]. About 15,000 hectares of wheat were destroyed, resulting in about 15% crop losses in Bangladesh [14]. The outbreak concerned crop scientists as it has the potential to extend further to major wheat-producing regions in neighboring South Asian countries and Africa due to similar climatic conditions [23]. Plant pathologists have cautioned that this disease is expected to disperse to India, Pakistan and China, that rank 2^nd^, 8^th^ and 1^st^, respectively, in the world for wheat production, [15, 24].

Current wheat blast disease management methods include the utilization of synthetic fungicides. Most of the synthetic fungicides are harmful to the environment and health of living species including humans [25-26]. Indiscriminate application of synthetic commercial fungicides for plant protection may also result in development of resistance in fungal population to fungicides [13, 17]. In Brazil and other South American countries, some *MoT* strains have developed resistance to strobilurin (QoI) and triazole fungicides [27, 28]. Nowadays, natural products that are environment-friendly with minimum toxicity to living organisms are gaining popularity as important ecologically suited alternative fungicides for protecting plants. Therefore, search for novel bioactive natural products against *MoT* is an urgently needed scientific endeavor.

The biological approach for plant disease management offers a better alternative to the control of wheat blast due to its low toxicity to humans and the environment. There is very little information available on the antagonistic effect of *Streptomyces* spp., and/or secondary metabolites derived from them to control wheat blast. We screened 150 natural compounds belonging to the class alkaloids, terpenoids, macrolides, macrotetrolides, tepenoids, and phenolics isolated from different plants and microorganisms for antagonistic activity against the wheat blast fungus *MoT* in our laboratory [21]. Among them, two most potential macrolides, oligomycin B and oligomycin F, previously extracted from the marine *Streptomyces* spp. [3-4] were selected for this study. The specific objectives of this study were to: (i) test the effect of oligomycin B and F on the inhibition of mycelial growth of *MoT*; (ii) examine their effect on conidiogenesis, conidial germination and subsequent morphological development; (iii) evaluate suppression of wheat blast using detached wheat leaves; and iv) compare the efficacy of these two oligomycins with a local standard fungicide product.

**Materials and methods**

**Chemicals**

Oligomycin B and oligomycin F (Fig. 1) were isolated from the marine bacteria, *Streptomyces* sp. strains B8496, B8739 and A171 [3-4]. These compounds were generously provided by Dr. Hartmut Laatsch of Georg-August University Goettingen, Germany. The fungicide Nativo® WG 75 (a combination of tebuconazole, 50% and trifloxystrobin, 25%) was purchased from Bayer Crop Science Ltd. Dhaka, Bangladesh.

**Fungal strain, growth media and plant materials**

The strain BTJP 4 (5) of *MoT* was isolated from blast infected spikelets of wheat cv. BARI Gom-24 (Prodip) in Jhenaidah, Bangladesh in 2016. A pure culture of a single colony isolate was conserved at 4°C on dry filter paper that was used for this research [14]. The isolate (BTJP 4) was re-cultured on Potato Dextrose Agar 42 g/L (PDA) by at 25^o^C for 7 days. Ten-day-old fungal cultures grown on PDA were washed in aseptic environment in a laminar flow hood with 500 ml of deionized distilled water for removing aerial mycelia and then kept at ambient room temperature (25-30^o^C) for 2-3 days to induce abundant conidia production [14, 29]. Conidia were scraped from the plates with a glass slide after adding 15 ml water in each plate. The conidial and mycelial suspensio was filtered by two layers of cheese cloth and adjusted to a concentration of 1 × 10^5^ conidia/ml. Conidial germination was visualized and counted under a compound microscope. Wheat blast susceptible cultivar BARI Gom-24 (Prodip) was used for the disease suppression bioassay on detached leaves from five-leaf stage seedlings [30].

**Inhibition of mycelial growth and morphological effects on hyphae**

A Modified disk diffusion technique [31] was used to determine hyphal growth suppression of Mot isolate BTJP by the oligomycins and the commercial fungicide, Nativo ® (5). A series of concentrations ranging from 0.005 to 2 μg / disk of the oligomycins and the fungicide Nativo ® WG75 were prepared by dissolving required amounts in ethyl acetate and water, respectively, followed by dilution with water. Filter-paper disks (Sigma-Aldrich Co., St. Louis, MO, USA) measuring nine-millimeter diameter were soaked with the test compounds. The treated discs were placed 2 cm from one side of 9 cm dia Petri dishes containing 20 ml PDA. Five-millimeter diameter mycelial plugs from actively growing seven-day-old PDA cultures of *Mot* were placed on the opposite side of the discs containing the test compounds. Filter paper discs treated with water served as a control. Inhibition of fungal growth was apparent 10 days of incubation. Plates were incubated at 25^o^C until the fungal colony fully covered the agar surface of the control. The experiment was replicated five times with each concentration and repeated twice. The fungal colony's radial growth was measured in centimeter with a ruler along with two perpendicular lines drawn on each plate's lower side. Data were recorded by the measuring inhibition zone created by test compounds and corresponding mycelial growth. Radial growth inhibition percentage (RGIP) (± standard error) [32] was calculated from mean values as:

$$RGIP (\%)=\frac{Radial growth in control plate-Radial growth in treated plate}{Radial growth of control} \times100$$

Hyphal morphology at the vicinity of compounds was observed under a ZEISS Primo Star microscope at 40x and 100x magnification. Images of inhibition zone were captured through a digital camera (Canon DOS 700D) and images of morphological features of hyphae were recorded by a camera (ZEISS Axiocam ERc 5s) attached to the microscope.

**Inhibition of conidiogenesis**

Stock solutions of each of the oligomycins were prepared in 10 μl of dimethyl sulfoxide (DMSO). Stock solution was then diluted with distilled water to obtain 5, 10 and 100 µg / ml concentrations. The final concentration of DMSO was never higher than 1% (v / v) in the final solution, which does not affect the hyphal growth or sporulation of *Mot*. Preparation of 5 ml fungicidal suspension of Nativo®WG75 at 5, 10 and 100 µg/ml concentrations was carried out by mixing the required amount of product in distilled water.the. Mycelium of a 10-day-old petri dish culture of MoT was washed to reduce nutrienst and induce conidiogenesis [14, 29]. Ten mm *MoT* mycelial agar blocks were treated with 50 μl of each compound and Nativo®WG75 at 5, 10 and 100 µg/ml and put into Nunc multiwell plates. The same amount of sterile water was applied on the mycelial block with 1% DMSO serving as a negative control. Treated mycelial agar blocks were incubated at 28^o^C with optimum humidity and lights. After 24 hours, conidiogenesis was observed with a Zeiss Primo Star microscope at 40x magnification and images captured with a Zeiss Axiocam ERc 5s There were five replications per treatment and the experiment was conducted five times.

**Inhibition of conidial germination and morphological changes of germinated conidia Conidial germination inhibition and morphological changes of germinated conidia by oligomycins**

Stock solutions of each oligomycin was prepared by dissolving 0.1 µg of the compound in 10 µl dimethyl sulfoxide (DMSO) followed by diluting the concentration. of each compound to 0.1µg/ml by adding distilled water. Nativo®WG75 solution was prepared with distilled water at 0.1 µg/ml to use as a positive control. Conidial germination assays were carried out following the protocol described by Islam and von Tiedemann [33]. For each treatment, a100 μl solution of 0.1 μg/ml was added directly to 100 μl of 1 × 10^5^ conidia of *Mo*to make a final volume of 200 μl containing 0.05 µg/ml test compound into a well of a 96-multiwell plate, The solution was mixed immediately with a glass rod and incubated at 25°C. Sterile water with 1% DMSO served as a control. The multiwell plate was incubated in a moisture chamber at 25 ^0^C for 6 h, 12 h and 24 h in the dark. A total of 100 conidia from each of five replicates were examined Zeiss Primo Star microscopeat 100x magnification. Percent germination of conidia and developmental differences of the germ tubes and appressoria were evaluated and images captured with a Zeiss Axiocam ERc 5s. Each treatment and time course was replicated five times and the experiments repeated five times. The percent of conidia germination (± standard error) was calculated from mean values as: CG % = (C – T)/C × 100; Where, CG = conidial germination, C = percentage of germinated conidia in control, and T = percentage of germinated conidia in treated samples.

**Development of wheat blast on detached wheat leaves**

Stock solutions of oligomycins B and F were prepared using dimethyl sulfoxide (DMSO). Then preparation of 5, 10 and 100 µg/ml concentrations of each compound was carried out in distilled water where the final DMSO concentration never exceeded 1%. Vativo WG 75 concentrations were 5, 10 and 100 µg/ml. Sterilized water with 1% DMSOserved as a negative control. Wheat leaves were separated from five-leaf stage seedlings and placed in plates lined with moist paper towels. Three 20-μl droplets of the freshly prepared test compounds at concentrations mentioned above were placed on three different spots of each leaf, and left for 15 minutes. Each spot was then inoculated with 1 μl conidial suspension containing 1 × 10^5^ *Mot* conidia followed by incubating dishes at 28^o^C under 100% relative humidity in dark for first 30 h , then 2 days in continuous light. The test was performed five times independently with 5 replicate samples. The resulting length of wheat blast lesions were measured from 3 leaves per experiment for each treatment and each concentration of compounds.

**Statistical analysis, experimental design/replications**

Experiments were performed using a completely randomized design (CRD) to determine biological activities of the pure oligomycin compounds compared to a standard fungicide. Data were analyzed by one-way ANOVA, and mean values were separated by the posthoc statistic of Tukey's HSD (honest significant difference). All statistical analyses were carriedoutby using SPSS (IBM SPSS statistics 16, Georgia, USA) and Microsoft Office Excel 2010 program package. Mean value ± standard error of 5 replications were used in Tables and Figures.

**Results**

**Inhibtion of mycelial growth and morphological effects on hyphae Effect of oligomycins on *MoT* mycelial growth and hyphal morphology**

Both oligomycins B and F (Fig 1) tested in this study and originally extracted from a *Streptomyces* species showed significant inhibition of *Mot* hyphal growth. BTJPon PDA (Fig 2). Between these two compounds, oligomycin F depicted stronger inhibition of hyphal growth (5). Mycelial growth by oligomycins B and F was 57.1 ± 1.3% and 73.9 ± 2.5%, respectively when both compounds were used at 2 µg/disk (Fig 3). The commercial fungicide Nativo® WG 75 had a higher inhibition capacity (81.9 ± 0.9% at 2 µg/ disk) than both oligomycin F and B.

**Fig 1. Structures of oligomycin B and F from (Dame et al., 2016)**

**Fig 2. Macroscopic and microscopic images of *in vitro* antifungal activity of oligomycin B, oligomycin F and the commercial fungicide nativo® WG75 against *M. oryzae* *Triticum* (*MoT*) at 2 µg/disk;** (a) Control, (b) Oligomycin B, (c) Oligomycin F, (d) Nativo® WG75. Bar = 50 μm.

**Fig 3. Inhibitory effects of oligomycin B, oligomycin F and the commercial fungicide Nativo® WG75 on hyphal growth of *Magnaporthe oryzae triticum* (*Mot*) in potato dextrose agar**. The data are the mean ± standard errors of five replicates for each concentration of the compound tested at a 5 % level based on the Tukey HSD (Honest Significance Difference) post-hoc statistic.

Both oligomycins inhibited *Mot*' mycelial growth in a dose dependent manner. Suppressive effects of oligomycin increased with increasing concentrations from 0.005 to 2 μg/disk reaching 74% for oligomycin F (Fig. 3). Suppression by oligomcin F was slightly lower than suppression by Nativo but higher than oligomycin B. Neither of the oligomycinshowed activity against *Mot* at concentration lower than 0.005 μg. Oligomycin F showed extensive inhibition of hyphal growth at 2 μg/disk (73.9 ± 2.5 %) followed by 1.5 μg/disk (67.6 ± 0.9 %) and 1 μg/disk (60.9 ± 2.5%) showing a positive correlation of suppression with an increase in concentration. The percent suppression by oligomycin B was 57.1 ± 1.3 %, 54.3 ± 1.3% and 53.3 ± 1.5%, at 2, 1.5 and 1 μg/disk, respectively. The minimum inhibitory concentrations (MICs) of oligomycin F and oligomycin B were 0.005 and 0.05 μg/disk, respectively (Fig 4). At the minimum inhibitory concentrations, hyphal growth inhibition was 11.4 ± 2.3% and 8.63 ± 1.3%, respectively for oligomycin F and B. Minimal inhibitory concentration of Nativo was 0.05 μg/disk (Fig 4), similar to oligomycin B although fungicide at higher concentration starting from 0.25 μg/disk superseded inhibition percentage at equivalent concentrations of oligomycins. It is interesting to note that at concentrations below 0.25 μg/disk, the inhibition of mycelial growth by oligomycin F was higher than that of the fungicide Nativo® WG 75 and this macrolide displayed inhibitory activity against *Mot* at about 10-fold lower concentration.

**Fig 4. Minimal Inhibitory Concentration (MIC) values of oligomycin B, oligomycin F and the fungicide Nativo® WG75 on hyphal growth of *Magnaporthe oryzae* *triticum* (*Mot*) in potato dextrose agar.**

Microscopic observations showed that untreated *Mot* hyphae had polar, tubular growth with smooth, branched, hyaline, plump, septate, and intact hyphae (Fig 2a). Hyphae treated with oligomycin B and F showed an increase in branch frequency per unit length of hyphae, destruction of regular growth with twisted ridges and corrugations, and irregular swelling of hyphal cells (Fig 2b and 2c). Nativo sowed a similar pattern of hyphal growthSimilar abnormality of *MoT* hyphae also occurred in the vicinity of Nativo ® WG75 disk that showed crystal appearance, loss of polar growth, erratic branching and swelling with twisted ridges and corrugations (Fig 2d). However, morphological changes of *MoT* by two oligomycins were slightly different from those occurred by the effect of commercial fungicide, Nativo®WG75 indicating a possible different mode of action.

**Inhibition of conidiogenesis**

Conidiogenesis of *Mot* was inhibited by both oligomycin compounds compared with abundant conidia formation in the untreated control after 24 h of incubation (Fig 5a). With the fungicide Nativo, inhibition of conidiogenesis increased with an increase of concentration from, 5 to 10 to 100 µg/ml (Fig 5d). Conidia formation was remarkably decreased by the oligomycins at 5 and 10 µg/ml concentrations compared to the untreated control (Fig 5b and 5c). Both compounds and the fungicide Nativo®WG75 completely inhibited conidiogenesis at 100 µg/ml (Fig. 5c and 5d). Microscopic observation also revealed the existence of broken mycelial tips with no sign of conidiophore and conidial growth on the surface of the agar block treated with macrolides at 100 μg / ml.

**Fig 5. Effects of oligomycin B, oligomycin F and the fungicide Nativo® WG75 on inhibition of conidiogenesis of *Magnaporthe oryzae triticum* in 96-multiwell plates at 5 µg/ml, 10 µg/ml, 100 µg/ml.** (a) Control, (b) Oligomycin B, (c) Oligomycin F, (d) Nativo® WG75. Bar = 50 μm.

**Inhibition of conidial germination and morphological changes of germinated conidia I**

Oligomycin B and oligomycin F at 0.05 μg / ml were used to determine the inhibition of conidial germination of *Mot* in multi-well plates. After 6, 12 and 24 h of incubation, the percent of germinated conidia was recorded (Table 1). After 6 h, all three treatments significantly reduced germination compared to the control  Germination was 100% in water, and 50.3±0.7% in plates treated with Nativo ® WG75. With Oligomycin B and F germination was 24 ± 0.9% and 53±0.4%) at 0.05 μg/ml, respectiviely.

**Table 1. Effects of oligomycins and and the fungicide Nativo on germination of conidia and morphology of germtubes and appressoria of *Magnaporthe oryzae triticum* at 0.05 µg/ml *in vitro***

| Compound | Time  (h) | Germination of conidia, and morphology of germ tubes and appressorial formation | |
| --- | --- | --- | --- |
|  |  | Germinated conidia (% ± SE^a^) | morphological change/developmental transitions in the treated conidia |
| Water | 0 | 0 ± 0e | No germination |
|  | 6 | 100 ± 0a | Germination with normal germ tube and normal appressoria |
|  | 12 | 100 ± 0a | Normal mycelial growth |
|  | 24 | 100 ± 0a | Normal mycelial growth |
| Oligomycin B | 0 | 0 ± 0e | No germination |
|  | 6 | 38.3 ± 0.7d | 24 ± 0.9% Short germ tube and 14.3± 0.7% conidia lysed |
|  | 12 | 24 ± 0.9d | 10.6 ± 0.7% Normal germtube, 8.4 ± 0.2% short and 5 ± 0.4% Abnormally elongated germ tube |
|  | 24 | 0 ± 0b | No appressoria, no mycelial growth |
| Oligomycin F | 0 | 0 ± 0e | No germination |
|  | 6 | 59 ± 0.8b | 53 ± 0.4% Short germ tube and 6 ± 0.8% conidia lysed |
|  | 12 | 53 ± 0.4b | 33.3 ± 0.5% Normal germ tube and 19.7 ± 0.2% abnormal branching at the tips |
|  | 24 | 0 ± 0b | No appressoria, no mycelial growth |
| Nativo | 0 | 0 ± 0e | No germination |
|  | 6 | 50.3 ± 0.7c | Germinated with a short germ tube |
|  | 12 | 50.3 ± 0.7c | Normal germ tube |
|  | 24 | 0 ± 0b | No appressoria; no mycelial growth |

^a^The data presented here are the mean value ± SE of three replicates in each compound. Means within the column followed by the same letter(s) are not significantly different from those assessed by Tukey's HSD (Honest Significance Difference) post-hoc (p ≤ 0.05). Conidia germination percent at different incubation times is not cumulative, rather at different time intervals.

**Inhibition of conidial germination and morphological changes of germinated conidia Developmental transitions of the treated conidia of *MoT* by oligomycins**

Conidial germination in water was 100% with normal germ tube development and mycelial growth at all incubation times (6 h, 12 h and 24 h) at 25°C in dark (Table 1, Fig 6a). The two oligomycins had adverse effects not only on conidial germination but also post-germination developmental processes with abnormal transitions from one step to the next at 0.05 µg/ml. Out of 38% of the germinated spores in the oligomycin B treatment, 24 ± 0.9% had short germ tubes and 14.3± 0.7% of the germ tubes lysed after 6 h of incubation. After 24 h of incubation in the same treatment, out of 24% germinated spores, 10.6 ± 0.7% were normal, 8.4 ± 0.2% had short germ tubes and 5 ± 0.4% had abnormally elongated germ tubes. After 24 h of incubation, no germinated conidia were found progressing to appressorial development and mycelial growth (Table 1, Fig 6b). In the presence of oligomycin F, 53 ± 0.4% of the conidia germinated with short germ tubes and 6 ± 0.8% conidia lysed after 6 h. Similar developmental abnormalities were found among the germinated conidia after 12 h of incubation with 33.3 ± 0.5% normal and 19.7 ± 0.2% abnormally branched germ tube formation, while no appressoria formation and mycelial growth recorded after 24 h (Table 1, Fig 6c). In the presence of Nativo, 50.3 ± 0.7% conidia germinated with normal germ tubes after 6h and 12 h, but no appressoria formed even until 24 h of incubation and thus no mycelial growth occurred (Table 1, Fig 6d). The findings revealed that these the oligomycins were capable of inducing major germ tube malformations, and suppressed formation of appressoria and hyphal growth of *Mot.* No conidial malformation was found in Nativo®WG75 treatment, but a slower growth of germ tubes was observed (Fig 6d).

**Fig 6. Time-dependent alterations in *Magnaporthe oryzae triticum* germination of conidia and subsequent morphological changes in the presence of oligomycin B, oligomycin F and the commercial fungicide Nativo® WG75.** Dose of oligomycins was 0.05 µg/ml. (a) Control, (b) Oligomycin B, (c) Oligomycin F, (d) Nativo® WG75. Bar = 10 μm.

**Development of wheat blast on detached wheat leaves** Application of the two oligomycins at the doses of 5, 10 and 100 µg/ml remarkably inhibited symptoms of wheat blast in detached wheat leaves, which were inoculated with *Mot* conidia. The average length of lesions recorded in the wheat leaves treated with oligomycin B were 6.3 ± 0.3 mm and 1.8±0.3 mm at 5µg/ml and 10 µg/ml, respectively (Fig 7A and 7B). With oligomycin F and Nativo®WG75, blast lesion lengths were 4 ± 0.3 mm and 2 ± 0.3 mm at 5 µg/ml (Fig. 7A, B). No blast symptoms were visible on the leaves when treated with oligomycin F and the fungicide Nativo®WG75 at 10 µg/ml and 100 µg/ml (Fig 7A and 7B). No visible blast lesions occurred treated bywith oligomycin B at 100 µg/ml. Leaves treated with water as a negative control developed typical blast lesions with an average length of 9.58 ± 0.2 mm (Fig 7A, 7B). These results show that suppression of blast on detached wheat leaves can be achieved with varying doses of oligomycins and that the level of suppression is dependent on the concentration. At lower rates, the oligomycins suppressed lesion development more than the fungicide Nativo. However, at higher rates with the oligomycins, the level of suppression was as good as the fungicide, especially for oligomycin F.

**Fig 7. Suppression of wheat blast symptoms with oligomycins at 5 µg/ml, 10 µg/ml and 100 µg/ml on detached wheat leaves inoculated with *Magnaporthe oryzae triticum* (A) blast lesions on treated and untreated wheat leaves (a) Inoculated water control, (b) Oligomycin B****+MoT inoculation, (c) Oligomycin F+MoT inoculation, (d) Nativo® WG75+MoT inoculation, (e) Non-inoculated, non-treated leaf; (B) Blast lesion lengths on detached wheat leaves treated with oligomycin B oligomycin F and Nativo fungicide. compared with water treatment control.** The data are the averages ± standard errors of at least five replicates for each dose of the tested compounds at p ≤ 0.05. Bars represent ± standard error.

**Discussion**

In this study, we found that two macrolides, oligomycin B and oligomycin F, isolated from *Streptomyces* spp. demonstrated extensive antifungal activities against the devastating wheat blast pathogen *MoT*. Bioassays demonstrated that both oligomycins strongly suppressed mycelial growth, conidiogenesis, germination of conidia and morphological changes of germinated conidia. They also inhibited wheat blast development as well or better than the commercial fungicide Notivo on detached leaves of wheat. Of the two oligomycins tested, biological activity of oligomycin F was superior than oligomycin B and in some cases, superior to the commercial fungicide Nativo® WG75. These findings indicate that suppression of, conidial germination, appressorial formation and mycelial growth by these macrolides are correlated with wheat blast disease suppression on inoculated leaves. Hyphal growth inhibition, conidial formation and germination of conidia of various fungi including the rice blast fungus (*MoO*) by a number of natural secondary metabolites from *in vitro* bioassays have been documented by many investigators [3, 11, 34-39]. To the best of our knowledge, this is the first report of suppression of the devastating wheat blast fungus by oligomycin B and F isolated from the *Streptomyces* spp. with the potential to be utilized for managing the disease in vivo.

Oligomycins are macrolide antibiotics that block the proton channel (F0 subunit) requisite for the oxidative phosphorylation of ADP to ATP due to inhibition of ATP synthase. [40]. While oligomycins have excellent biological properties, only a few studies have so far focused on the development of plant disease protection products from these macrolides. Interestingly, our results also had revealed that the efficacy of oligomycin F in controlling wheat blast fungus was 10-fold stronger than the commercial fungicide Nativo ® WG75. This may not be true if you make the comparison at the same active ingredient, however.

One of the noteworthy findings from the present study is the induction of swelling on the *MoT* hyphae by these macrolides (Fig 2b and 2c), which is often considered as a reliable mode of inhibitory action of a compound against the normal growth and development of a fungal pathogen. We tested a range of concentrations of these compounds from 0.005 to 2 µg /disk, and our results showed that swelling in hyphae increased with increasing concentrations of the oligomycins. Swelling in various fungal hyphae has been reported earlier by polyoxin B [41], fengycin [37-39] and tensin [42]. Morphological changes like extensive branching and swelling of hyphae of an the oomycete pathogen *Aphanomyces cochlioides* by phloroglucinols extracted from *Pseudomonas fluorescence* or xanthobaccin A isolated from *Lysobacter* sp. SB-K88 have been documented [43-46]. According to a report by Kim et al. [11], oligomycin A from *Streptomyces libani*, significantly inhibited mycelial growth of *Magnaporthe grisea, Botrytis cinerea, Colletotrichum lagenarium, Cylindrocarpon destructans, Cladosporium cucumerinum and Phytophthora capsici.* So far, this is the first report of swollen-like structures development in hyphae by oligomycins toward the destructive phytopathogen, *MoT*. Further investigation is needed to understand the detailed modes of action of these macrolides towards suppression of the phytopathogen *Mot*.

Conidia are the infecting propagule by which most pathogenic fungi invade plants and the process by which conidia are formed is known as conidiogenesis [35, 47]. The more conidia a fungal pathogen produces the more its potential to destroy a plant, which is very significant in case of an economically important cereal crop like wheat. Inhibition of conidiogenesis and conidial germination reduces the chance of secondary infection. As such compounds that are found to inhibit these processes are great candidate for downstream application as plant protection products given additional work is done. A few interesting findings of this study showed that both oligomycin B and F not only strongly suppressed conidiogenesis (Fig 5), but also inhibited germination of conidia and further morphological advancement of germ tube towards hyphal growth (Table 1, Fig 6). Results from the bioassay showed that wheat leaves prophylactically treated with these macrolides at 5, 10 and 100 µg/ml had restricted fungal growth and very limited disease development by *MoT* conidia. Other novel and interacting phenomena observed in this study included lysis of conidia, irregular branching at the tip of conidial germ tubes, and abnormally elongated hypha-like germ tubes (Fig 6b and 6c) due to the interaction of MoT conidia with the macrolides. Similar phenomenon was observed by Dame et al. [4] who reported that oligomycin A, B and F from a marine *Streptomyces* could induce lysis of zoospores of the pathogen *Plasmopara viticola* which causes grapevine downy mildew. Homma et al. [48] reported that lecithin induced abnormal branching at the conidial germ tube tips and inhibited appressoria formation of rice blast fungus. Islam and Fukushi [46] have observed that cystospores of *A. cochlioides* produced in the presence of diacylphloroglucinol (DAPG) subsequently germinated with hyperbranched germ tubes. Modes of action and mechanism of inhibition of conidiogenesis, germination and formation of appressoria of the *MoT* conidia by oligomycins has not been previously reported. However, our findings of developmental abnormalities in *MoT* conidia suggest that these compounds might influence gene expression upstream of melanin synthesis and block intracellular signaling or signal transduction pathways, which regulate appressoria formation [50]. The effect of these bioactive natural compounds on the expression of germination of conidia and formation of appressorium related genes in *MoT* should be focused in future studies.

ATPase has recently appeared as a promising molecular target for developing new therapeutic options for a variety of diseases. ATP synthase is thought to be one of the oldest and most conserved enzymes in the molecular world and has a complex structure with the probability of inhibition by a range of inhibitors [51]. It is of great scientific relevance that oligomycins are macrolide antibiotics that impede ATP production by influencing oxidative phosphorylation in mitochondria [52]. Oligomycin comprises a 26-membered α, β-unsaturated lactone with a conjugated diene fused to a bicyclic spiroketal ring system. Their mode of action includes the decoupling of mitochondrial ATPase F0 and F1 factors responsible for promoting the transfer of proton via the inner mitochondrial membrane [53]. The enzymatic complex F0F1 ATP synthase may be considered as a target for antifungal and anti-tumor or anti-infection therapy [54]. Oligomycins display a number of important biological activities including mitochondrial ATPase inhibition, strong antifungal, anti-actinobacterial and anti-tumor effects that have been reported [9, 1, 54]. These natural products are among the strongest selective agents in the cell line; they interrupt P-glycoprotein activity and induce apoptosis in doxorubicin-resistant HepG2 cells [55]. Oligomycins have a variety of isomers called oligomycin A through G. These are particularly relevant to the disruption of mitochondrial metabolism [10]. Moreover, elucidation of structure has provided new horizons for developing new ATP synthase-directed agents with possible therapeutic effects [51]. The first reports of chemical modification of oligomycin A have already been documented by Lysenkova et al. [56]. New compounds also showed efficacy against *Candida albicans, Aspergillus niger* and *Cryptococcus humicolus*, with other biological properties similar to those of oligomycin A, but with less cytotoxic effects. During germination, conidia might need a constant energy (ATP) supply from the internal energy reserve of the cells [57]. Therefore, a plausible explanation for the suppression of hyphal growth and conidia germination of *MoT* depicted in this study is likely to be associated with the ATP synthesis inhibition in mitochondria due to the effects of oligomycins. More studies are required to determine the precise structure-activity relationships of these oligomycins, which may make it possible to synthesize a more active oligomycin as an effective agrochemical against *MoT*.

A hallmark finding of this study is that application of both macrolides significantly inhibited blast disease development in detached leaves of wheat (Fig 7), which can be explained as consequences of the events in the in vitro results of developmental inhibition of the pathogen by the oligomycins. In this study, wheat leaves treated with oligomycin B and F had shorter lesion lengths than the untreated control (Fig 7). Many of the lesions in treated leaves were small brown in color with pinhead-sized specks (scale 1) to small, roundish to slightly elongated infecting <10% of wheat leaf area (scale 5). In contrast, water treated control leaves had typical blast lesions infecting 26-50% wheat leaf area (scale 7) corresponding to scale 9 of blast disease assessment scale provided by the IRRI Standard Evaluation System [58]. However, no blast lesions were visible on the leaves treated with the oligomycin compounds and Nativo ® WG75 at the highest concentration (Fig 7). Nativo ® WG75 is a systemic wide-spectrum commercial fungicide that we used as a positive control. Interestingly, the antifungal effect of oligomycins on the inhibition of *MoT* fungus was found equivalent or stronger than that of the fungicide. Tebuconazole and trifloxystrobin are two main active ingredients of Nativo ® WG75. Tebuconazole is known as a demethylase inhibitor (DMI), which is a systemic triazole fungicide. This active ingredient of the fungicide inhibits ergosterol biosynthesis, which is a major component of the plasma membrane of certain fungi and needed for growth and further development of the fungus [59]. Trifloxystrobin is a strobilurin fungicide that interferes with the respiration of plant pathogenic fungi by preventing energy production in mitochondria, thereby inhibiting germination of fungal conidia [60]. The mechanism of disease suppression by the oligomycins used in this study have some differences compared with Nativo ® WG75 although similar disease suppression effect has been obtained. Further study is needed to elucidate the underlying mechanism of wheat blast disease suppression by the oligomycin B and F. Furthermore, a field trial of the oligomycins in controlling wheat head infection is needed before considering them as effective fungicides against the wheat blast.

Despite the significant potency as antifungal compounds, little has information available on the effectiveness of oligomycins as agricultural fungicides. The shorter residual effect of oligomycin can be important in the reduction of deleterious effects on humans and the environment, considering that sufficient efficacy in the management of plant diseases is sustained [11]. In the *in vivo* experiments for determining the efficacy of oligomycin A against a range of plant diseases such as *Phytophthora* blight of pepper plants, anthracnose on cucumber leaves, and rice leaf blast, this compound provided substantial protection against certain plant pathogen infections when treated at 500 μg/mL. Treatment of host plant at this concentration in some cases provided extended protection for up to a month [11]. Oligomycin A was found the most active anti-filamentous fungi analogue among antibiotics in the oligomycin family [1, 61, 62]. As oligomycin F is the immunosuppressive homolog of oligomycin A [3], and oligomycin B is a stable natural product [63], these macrolides have the potential to be the leading compounds for the production of agrochemicals against the cereal killer *MoT*.

The morphological changes induced by these macrolides in *MoT* are similar to several active, selective and cell-permeable antifungal secondary metabolites, like chelerythrine chloride, staurosporin, polyoxin A, polyoxin B and polyoxin D. Chelerythrine chloride and staurosporin are inhibitors of protein kinase C that have induced swelling of hyphal subapical areas, inhibited spore germination, and inhibited formation of appressoria of plant pathogenic fungi by blocking ATP binding to kinase [64-67]. Polyoxins are well-known as inhibitors of cell wall biosynthesis that suppress formation of germ tubes and appressoria [41, 68-70]. Efficacy of oligomycins tested in this study were also comparable to several commercially available fungicides such as blasticidin-S, kasugamycin, streptomycin and tricyclazole, which demonstrated strong antifungal efficacy against many phytopathogenic fungi like rice blast fungus *M. oryzae Oryzae* (*MoO*) [11, 71-73]. The typical mode of action of these fungicides are inhibition of protein synthesis, microcondrial ATPase, cell wall and melanin biosynthesis that result in the inhibition of fungal mycelial growth, germination of spore and formation of appressoria.

Frequent application of commercial fungicides with site-specific modes of action, like strobilurin (QoI) and triazole, has resulted in the widespread occurrence of resistant mutant species in *MoT* [27, 28]. Resistance development in fungal population against fungicides prompted search for new, effective antifungal agents with alternate mode of action to protect wheat plants against this dangerous phytopathogenic fungus. Results from this study pertaining to the inhibitory ability of these macrolides should motivate agrochemical companies to consider these as candidates for commercial products with novel modes of action against the notorious wheat blast fungus.

**Conclusion**

Our findings for the first time, have shown that the two macrolides, oligomycin B and F from *Streptomyces* spp., suppressed hyphal growth and asexual development of *Mot* t, and inhibited wheat blast tdevelopment on detached leaves of wheat. Field assessment of these macrolides is required to evaluate these metabolites as effective fungicides against wheat blast t. Further research is also required to understand the mode of action and the structure-activity relations among oligomycins A-G against the devastating wheat killer, *M. oryzae triticum*.

**Funding**

This work was funded by the Krishi Gobeshona Foundation (KGF), Bangladesh through a coordinated project No. KGF TF 50-C/17 to Tofazzal Islam of the Institute of Biotechnology and Genetic Engineering of BSMRAU, Bangladesh.

**Conflict of Interest Statement**

The authors declare that there is no conflict of interest.

**Ethics statement**

There is no ethical issues in the experiment of this manuscript.

**Author contributions**

**Conceptualization:** Tofazzal Islam

**Data curation:** Tofazzal Islam

**Formal analysis:** Moutoshi Chakraborty, Nur Uddin Mahmud

**Investigation:** Moutoshi Chakraborty, Nur Uddin Mahmud

**Resources:** Moutoshi Chakraborty, Nur Uddin Mahmud, Abu Naim Md. Muzahid, S. M. Fajle Rabby

**Methodology:** Moutoshi Chakraborty, Nur Uddin Mahmud, Abu Naim Md. Muzahid, S. M. Fajle Rabby

**Project administration:** Tofazzal Islam

**Software:** Moutoshi Chakraborty, Nur Uddin Mahmud

**Supervision:** Tofazzal Islam

**Funding acquisition:** Tofazzal Islam

**Visualization:** Tofazzal Islam

**Validation:** Tofazzal Islam.

**Writing – original draft:** Moutoshi Chakraborty, Nur Uddin Mahmud

**Writing – review & editing:** Tofazzal Islam

**Acknowledgements**

The authors are thankful to the Krishi Gobeshona Foundation (KGF), Bangladesh (KGF-TF 50-C/17) for funding this work. The funders had no role in study design, data collection and analysis, decision to publish, or preparation of the manuscript. The authors are also thankful to Dr. Hartmut Laatsch of Georg-August University Goettingen, Germany, for kindly providing the oligomycins for this research. Our sincere thanks are due to Dr. Mahfuzur Rahman and Tahsin Islam Sakif of West Virginia University, USA for linguistic editing of this manuscript.

**References**

1. Masamune S, Sehgal JM, van Tamelen, EE, Strong FM, Peterson WH. Separation and preliminary characterization of oligomycins A, B and C. J Am Chem Soc. 1958; 80: 6092–95. [doi: 10.1021/ja01555a049](https://doi.org/10.1021/ja01555a049)
2. Yamazaki M, Yamashita T, Harada T, Nishikiori T, Saito S, Shimada N, Fujii, A. 44-homooligomycins A and B, new antitumor antibiotics from *Streptomyces bottropensis* producing organism, fermentation, isolation, structure elucidation and biological properties. J Antibiot. 1992; 45: 171–79. [doi: 10.7164/antibiotics.45.171](https://doi.org/10.7164/antibiotics.45.171)
3. Laatsch H, Kellner M, Wolf G, Lee YS, Hansske F, Konetschny-Rapp S, et al. Oligomycin F, a new immunosuppressive homologue of oligomycin A. J Antibiot. 1993; 46: 1334–41. [doi: 10.7164/antibiotics.46.1334](https://doi.org/10.7164/antibiotics.46.1334)
4. Dame ZT, Islam MT, Helmke E, von Tiedemann A, Laatsch H. Oligomycins and pamamycin homologs impair motility and induce lysis of zoospores of the grapevine downy mildew pathogen, Plasmopara viticola. FEMS Microbiol. Lett. 2016; 363(16) fnw167: 1-6. [doi: 10.1093/femsle/fnw167](https://doi.org/10.1093/femsle/fnw167)
5. Newitt JT, Prudence SMM, Hutchings MI, Worsley SF. Biocontrol of cereal crop diseases using *Streptomycetes*. Pathogens. 2019; 8: 78. [doi: 3390/pathogens8020078](https://doi.org/3390/pathogens8020078).
6. Ōmura, S. Microbial metabolites: 45 years of wandering, wondering and discovering. Tetrahedron. 2011; 67 (35): 6420–59. [doi: 10.1016/j.tet.2011.03.117](https://doi.org/p10.1016/j.tet.2011.03.117)
7. Enomoto Y, Shiomi K, Matsumoto A, Takahashi Y, Iwai, Y, Harder A, et al. Isolation of a new antibiotic oligomycin G produced by Streptomyces sp. WK-6150. J Antibiot. 2001, 54: 308–13. [doi: 10.7164/antibiotics.54.308](https://doi.org/10.7164/antibiotics.54.308)
8. Salomon AR, Voehringer DW, Herzenberg LA, Khosla C. Apoptolidin, a selective cytotoxic agent, is an inhibitor of F0F1-ATPase. Chem Biol. 2001; 8: 71–80. [doi: 10.1016/S1074-5521(00)00057-0](https://doi.org/10.1016/S1074-5521(00)00057-0)
9. Kobayashi K, Nishino C, Ohya J, Sato S, Mikawa T, Shiobara Y, et al. Oligomycin E, a new antitumor antibiotic produced by *Streptomyces* sp. MCI-2225. J Antibiot. 1987; 40, 1053–57. [doi: 10.7164/antibiotics.40.1053](https://doi.org/10.7164/antibiotics.40.1053)
10. Shin YK, Yoo BC, Chang HJ, Jeon E, Hong SH, Jung MS, et al. Down-regulation of mitochondrial F1F0-ATP synthase in human colon cancer cells with induced 5-fluorouracil resistance. Cancer Res. 2005; 65(8): 3162-70. [doi: 10.1158/0008-5472.CAN-04-3300](https://doi.org/10.1158/0008-5472.CAN-04-3300)
11. Kim BS, Moon SS, Hwang BK. Isolation, identification, and antifungal activity of a macrolide antibiotic, oligomycin A, produced by *Streptomyces libani*. Can J Bot. 1999; 77: 850–58. [doi: 10.1139/b99-044](https://doi.org/10.1139/b99-044)
12. Igarashi S, Utiamada CM, Igarashi LC, Kazuma AH, Lopes RS. Pyriculariaemtrigo. 1. Ocorrência de Pyricularia sp. no estado do Paraná. Fitopatol. Bras. 1986; 11: 351–52.
13. Kohli MM, Mehta YR, Guzman E, Viedma L, Cubilla LE. *Pyricularia* blast-a threat to wheat cultivation. Czech J Genet Plant Breed. 2011; 47: 130-34.
14. Islam MT, Croll D, Gladieux P, Soanes DM, Persoons A, Bhattacharjee P, et al., Emergence of wheat blast in Bangladesh was caused by a South American lineage of *Magnaporthe oryzae*. BMC Biol. 2016; 14, 84. [doi: 10.1186/s12915-016-0309-7](https://doi.org/10.1186/s12915-016-0309-7)
15. Islam MT, Kim KH, Choi J. Wheat blast in Bangladesh: the current situation and future impacts. Plant Pathol.. J. 2019; 35(1): 1–10. [doi: 10.5423/PPJ.RW.08.2018.0168](https://doi.org/10.5423/PPJ.RW.08.2018.0168)
16. Tufan HA, McGrann GRD, Magusin A, Morel JB, Miche L, Boyd LA. Wheat blast: histopathology and transcriptome reprogramming in response to adapted and nonadapted *Magnaporthe* isolates. New Phytol. 2009; 184(2): 473-84.
17. Ceresini PC, Castroagudín VL, Rodriguez FA, Rios JA, Aucique-Pérez CE, Moreira SI, et al. Wheat blast: from its origin in South America to its emergence as a global threat. Mol Plant Pathol. 2019; 20(2): 155-72. doi: 10.1111/mpp.12747
18. Wilson RA, Talbot NJ. Under pressure: investigating the biology of plant infection by *Magnaporthe oryzae*. Nat Rev Microbiol. 2009; 7(3): 185–95. doi:10.1038/nrmicro2032.
19. Inoue K, Suzuki T, Ikeda K, Jiang S, Hosogi N, Hyon GS, et al. Extracellular matrix of *Magnaporthe oryzae* may have a role in host adhesion during fungal penetration and is digested by matrix metalloproteinases. J Gen Plant Pathol. 2007; 73: 388–98. [doi: 10.1007/s10327-007-0048-2](https://doi.org/10.1007/s10327-007-0048-2)
20. Urashima AS, Hashimoto Y, Le Don D, Kusaba M, Tosa Y, Nakayashiki, et al. Molecular analysis of the wheat blast population in Brazil with a homolog of retrotransposon MGR583. Jpn J Phytopathol. 1999; 65: 429–36. [doi: 10.3186/ jjphytopath.65.429](https://doi.org/10.3186/%20jjphytopath.65.429)
21. Chakraborty M, Mahmud NU, Gupta DR, Tareq FS, Shin HJ, Islam T. Inhibitory effects of linear lipopeptides from a marine *Bacillus subtilis* on the wheat blast fungus *Magnaporthe oryzae Triticum*. Front Microbiol. 2020; 11(665): 1-14. [doi: 10.3389/fmicb.2020.00665](https://doi.org/10.3389/fmicb.2020.00665)
22. Callaway E. Devastating wheat fungus appears in Asia for first time. Nature. 2016; 532: 421-422. doi: [10.1038/532421a](https://doi.org/10.1038/532421a)
23. CIMMYT. Wheat Blast Disease: A Deadly and Baffling Fungal Foe. International Maize and Wheat Improvement Center; Texcoco, Mexico. 2016.
24. Mundi. Agricultural production, supply, and distribution: wheat production by country in 1000 MT. 2016.
25. Nannipieri P. The potential use of soil enzymes as indicators of productivity, sustainability and pollution. In Soil biota-management in sustainable farming systems; Pankhurst, C. E., Doube, B. M., Gupta, V. V. S. R., Grace, P. R., Eds.; CSIRO: Collingwood, Australia, 1994; pp 33−140.
26. Suprapta DN. Potential of microbial antagonists as bio-control agents against plant pathogens. J ISSAAS. 2012; 18: 1−8.
27. Castroagudín VL, Ceresini PC, De Oliveira SC, Reges JTA, Maciel JLN, Bonato ALV, et al. Resistance to QoI fungicides is widespread in Brazilian populations of the wheat blast pathogen *Magnaporthe oryzae*. Phytopathology 2015; 105: 284–94. [doi: 10.1094/PHYTO-06-14-0184-R](https://doi.org/10.1094/PHYTO-06-14-0184-R)
28. Dorigan AF, Carvalho GD, Poloni NM, Negrisoli MM, Maciel JLN, Ceresini PC. Resistance to triazole fungicides in *Pyricularia* species associated with invasive plants from wheat fields in Brazil. Acta Sci Agron. 2019; 41(1): 39332. [doi:](https://doi.org/) 10.4025/actasciagron. v41i1.39332
29. Urashima AS, Igarashi S, Kato H. Host range, mating type, and fertility of *Pyricularia grisea* from wheat in Brazil. Plant Dis. 1993; 77(12): 1211-16. doi: [10.1094/PD-77-1211](https://www.researchgate.net/deref/http%3A%2F%2Fdx.doi.org%2F10.1094%2FPD-77-1211?_sg%5B0%5D=5qEqOXyXGRdRKf_MQsWorzh4IhUnO_FPF0Dp6iyKf0MwMRymjcVcnXQ0uoLgvuxewH33cujGiisMWT4l-ICR91Q5ig.gPnlJoz-bGYSyA06Ly25V-vu4sicslNl2GY0MJbpJgH1fKeSg4UOpIXDqWU_I6IWcnroINrWrDE0rUqtAfy2wA)
30. He Y, Zhu M, Huang J, Hsiang T, Zheng L. Biocontrol potential of a *Bacillus subtilis* strain BJ-1 against the rice blast fungus *Magnaporthe oryzae*. Can J Plant Pathol. 2019; 41(1): 47-59. [[doi:](https://doi.org/) 10.1080/07060661.2018.1564792](https://doi.org/10.1080/07060661.2018.1564792)
31. Bauer AW, Kirby WM, Sherris JC, Turck M. Antibiotic susceptibility testing by a standardized single disk method. Am J Clin Pathol. 1966; 45(4): 493–96. doi: 10.1093/ajcp/45.4_ts.493
32. Riungu GM, Muthorni JW, Narla RD, Wagacha JM, Gathumbi JK. Management of *Fusarium* head blight of wheat and deoxynivalenol accumulation using antagonistic microorganisms. Plant Pathol. J. 2008; 7: 13-9. doi: [10.3923/ppj.200](http://dx.doi.org/10.3923/ppj.2008.13.19)
33. Islam MT, von Tiedemann A. 2,4-Diacetylphloroglucinol suppresses zoosporogenesis and impairs motility of Peronosporomycete zoospores. World J Microb Biot. 2011; 27(9): 2071–79. [doi:](https://doi.org/) 10.1007/s11274-011-0669-7
34. Romero D, de Vicente A, Olmos JL, Davila JC, Perez-Garcia A. Effect of lipopeptides of antagonistic strains of *Bacillus subtilis* on the morphology and ultrastructure of the cucurbit fungal pathogen *Podosphaera fusca*. J Appl Microbiol. 2007; 103: 969–76. doi: [10.1111/j.1365-2672.2007.03323.x](https://doi.org/10.1111/j.1365-2672.2007.03323.x)
35. Kopecká M, Ilkovics L, Ramíková V, Yamaguchi M. Effect of cytoskeleton inhibitors on conidiogenesis and capsule in the long neck yeast *Fellomyces* examined by scanning electron microscopy. Chemo. 2010; 56(3): 197–202. doi: 10.1159/000316330
36. Tang QY, Bie XM, Lu ZX, Lv FX, Tao Y, Qu XX. Effects of fengycin from *Bacillus subtilis* fmbJ on apoptosis and necrosis in *Rhizopus stolonifer*. J Microbiol. 2014; 2: 675–680. doi: 10.1007/s12275-014-3605-3
37. Gond SK, Bergen MS, Torres MS, White JFJr. Endophytic *Bacillus* spp. produce antifungal lipopeptides and induce host defence gene expression in maize. Microbiol Res. 2015; 172: 79–87. [doi:](https://doi.org/) 10.1016/j.micres.2014.11.004
38. Liao JH, Chen PY, Yang YL, Kan SC, Hsieh FC, Liu YC. Clarification of the antagonistic effect of the lipopeptides produced by *Bacillus amyloliquefaciens* BPD1 against *Pyricularia oryzae* via In Situ MALDI-TOF IMS Analysis. Molecules 2016; 21(12): 1670. [doi: 10.3390/molecules](https://doi.org/10.3390/molecules) 21121670
39. Zhang L, Sun C. Fengycins, cyclic lipopeptides from marine *Bacillus subtilis* strains, kill the plant-pathogenic fungus *Magnaporthe grisea* by inducing reactive oxygen species production and chromatin condensation. Appl Environ Microbiol. 2018; 84: e00445-18. doi: 10.1128/AEM.00445-18
40. Jastroch M, Divakaruni AS, Mookerjee S, Treberg JR, Brand MD. Mitochondrial proton and electron leaks. Essays Biochem. 2010; 47(1): 53–67. [doi: 10.1042/](https://doi.org/10.1042/) bse0470053
41. Isono K, Nagatsu J, Kawashima Y, Suzuki S. Studies on polyoxins, antifungal antibiotics. Agric Biol Chem. 1965; 29(9): 848–54. [doi: 10.1080/00021369.1965. 10858475](https://doi.org/10.1080/00021369.1965.%2010858475)
42. Nielsen TH, Thrane C, Christophersen C, Anthoni U, Sørensen J. Structure, production characteristics and fungal antagonism of tensin – a new antifungal cyclic lipopeptide from *Pseudomonas fluorescens* strain 96.578. J Appl Microbiol. 2000; 89: 992-1001. doi: 10.1046/j.1365-2672.2000.01201.x
43. Islam MT, Hashidoko Y, Deora A, Ito T, Tahara S. Suppression of damping-off disease in host plants by the rhizoplane bacterium *Lysobacter* sp. strain SB-K88 is linked to plant colonization and antibiosis against soilborne Peronosporomycetes. Appl Environ Microb. 2005; 71: 3786–3796. [doi:](https://doi.org/) 10.1128/AEM.71.7.3786-3796.2005
44. Islam MT. Disruption of ultrastructure and cytoskeletal network is involved with biocontrol of damping-off pathogen *Aphanomyces cochlioides* by *Lysobacter* sp. strain SB-K88. Biol Control 2008; 46: 312–21. <https://doi.org/>10.1016/j.biocontrol.2008.02.006
45. Islam MT. Mode of antagonism of a biocontrol bacterium *Lysobacter* sp. SB-K88 toward a damping-off pathogen *Aphanomyces cochlioides*. World J. Microb. Biot. 2010; 26: 629-37. [doi: 10.1007/s11274-009-0216-y](https://doi.org/10.1007/s11274-009-0216-y)
46. Islam MT, Fukushi Y. Growth inhibition and excessive branching in *Aphanomyces cochlioides* induced by 2,4-diacetylphloroglucinol is linked to disruption of filamentous actin cytoskeleton in the hyphae. World J Microb Biot. 2010; 26: 1163-70. [doi:](https://doi.org/) 10.1007/s11274-009-0284-z
47. Ohara T. REN1 is required for development of microconidia and macroconidia, but not of Chlamydospores, in the plant pathogenic fungus *Fusarium oxysporum*. Genet. 2004; 166(1): 113–24. [doi: 10.1534/genetics.166.1.113](https://doi.org/10.1534/genetics.166.1.113)
48. Homma Y, Takahashi H, Arimoto Y. Studies on the mode of action of soybean lecithin. JJP. 1992; 58(4): 514-21. [doi: 10.3186/jjphytopath.58.514](https://doi.org/10.3186/jjphytopath.58.514)
49. Carver TLW, Roberts PC, Thomas BJ, Lyngkær MF. Inhibition of *Blumeria graminis* germination and germling development within colonies of oat mildew. Physiol Mol Plant Pathol. 2001; 58(5): 209–28. doi:10.1006/pmpp.2001.0330
50. Hall AA, Gurr SJ. Initiation of appressorial germ tube differentiation and appressorial hooking: distinct morphological events regulated by cAMP-signalling in *Blumeria graminis* f. sp. *hordei*. Physiol Mol Plant Pathol. 2000; 56: 39-46. [doi: 10.1006/pmpp.1999.0246](https://doi.org/10.1006/pmpp.1999.0246)
51. Neupane P, Bhuju S, Thapa N, Bhattarai HK. ATP Synthase: Structure, Function and Inhibition. Biomol Con. 2019; 10(1): 1–10. doi: 10.1515/bmc-2019-0001
52. Smith RM, Peterson WH, McCoy E. Oligomycin, a new antifungal antibiotic. Antibiot Chemother. 1954; 4: 962–70.
53. Pagliarani A, Sesci S, Ventrella V. Modifiers of the oligomycin sensitivity of the mitochondrial F1F0-ATPase. Mitochondrion 2013; 13: 312–19. [doi: 10.1016/j.mito.2013.04.005](https://doi.org/10.1016/j.mito.2013.04.005)
54. Alekseeva MG, Elizarov SM, Bekker OB, Lubimova IK, Danilenko VN. F0F1ATP synthase of streptomycetes: modulation of activity and oligomycin resistance by protein Ser/Thr kinases. Biol Membrany. 2009; 26: 41–9. [doi: 10.1134/ S1990747809010036](https://doi.org/10.1134/%20S1990747809010036)
55. Li YC, Fung KP, Kwok TT, Lee CY, Suen YK, Kong SK. Mitochondria-targeting drug oligomycin blocked P-glycoprotein activity and triggered apoptosis in doxorubicin-resistant HepG2 Cells. Chemotherapy 2004; 50: 55–62. doi: [10.1159/000077803](https://doi.org/10.1159/000077803)
56. Lysenkova LN, Turchin KF, Danilenko VN, Korolev AM, Preobrazhenskaya MN. The first examples of chemical modification of oligomycin A. J Antibiot. 2009; 63(1): 17–22. doi: 10.1038/ja.2009.112
57. Bimpong CE. Changes in metabolic reserves and enzyme activities during zoospore motility and cyst germination in *Phytophthora* *palmivora*. Can J Bot. 1975; 53: 1411–16. [doi: 10.1139/b75-170](https://doi.org/10.1139/b75-170)
58. IRRI. Standard Evaluation System for Rice. 4th Edn., International Rice Research Institute (IRRI), Los Banos, Philippines, 1996; Pages: 52.
59. Pring RJ. Effects of triadimefon on the ultrastructure of rust fungi infecting leaves of wheat and broad bean (*Vicia faba*). Pestic Biochem Phys. 1984; 21(1): 127–37.
60. Sauter H, Steglich W, Anke T. Strobilurins: evolution of a new class of active substances. *Angew.* Chem Int Ed Engl. 1999;  38: 1328-49. [doi: 10.1002/(SICI)1521-3773(19990517)38:10<1328::AID-ANIE1328>3.0.CO;2-1](https://doi.org/10.1002/(SICI)1521-3773(19990517)38:10%3c1328::AID-ANIE1328%3e3.0.CO;2-1)
61. Marty EW, McCoy E. The chromatographic separation and biological properties of the oligomycins. Antibiot Chemother. 1959; 9: 286–93.
62. Sakagami Y, Ueda A, Yamabayashi S, Tsurumaki Y, Kumon S. A new antibiotic, hondamycin. I. Isolation and characterization. J Antibiot. 1969; 22: 521–27. doi: [10.7164/antibiotics.22.521](https://doi.org/10.7164/antibiotics.22.521)
63. Lardy HA, Witonsky P, Johnson D. Antibiotics as tools for metabolic studies. IV. Comparative effectiveness of oligomycins A, B, C, and rutamycin as inhibitors of phosphoryl transfer reactions in mitochondria. Biochem. 1965; 4: 552–54.
64. Agae Y, Magae J. Effect of staurosporine on growth and hyphal morphology of *Pleurotus ostreatus*. J Gen Microbiol. 1993; 139: 161-64. doi: 10.1099/00221287-139-1-161
65. Penn TJ, Wood ME, Soanes DM, Csukai M , Corran AJ, Talbot NJ. Protein kinase C is essential for viability of the rice blast fungus *Magnaporthe oryzae*. Mol Microbiol. 2015; 98(3): 403–19. doi: 10.1111/mmi.13132
66. Wei Q, Zhao M, Li X. Extraction of chelerythrine and its effects on pathogenic fungus spore germination. Phcog Mag. 2017; 13; 600-606. doi: 10.4103/pm.pm_545_16
67. Sugahara A, Yoshimi A, Shoji F, Fujioka T, Kawai K, Umeyama H, et al. Novel antifungal compound Z-705 specifically inhibits protein kinase C of filamentous fungi. Appl Environ Microbiol. 2019; 85: e02923- e02918. doi: 10.1128/AEM.02923-18
68. Endo A, Kakiki K, Misato J. Mechanism of action of the antifungal agent polyoxin D. J Bacteriol. 1970; 104: 189-96.
69. Barka EA, Vatsa P, Sanchez L, Gaveau-Vaillant N, Jacquard C, Meier-Kolthoff JP, et al. Taxonomy, physiology, and natural products of actinobacteria. Microbiol Mol Biol Rev. 2016; 80: 1–43. https://doi.org/10.1128/MMBR.00019-15
70. Copping LG, Duke SO. Natural products that have been used commercially as crop protection agents. Pest Manag Sci. 2007; 63(6): 524–554. doi: 10.1002/ps.1378
71. Yamaguchi I. Fungicides for control of rice blast disease. J Pest Sci. 1982; 7: 307–16.
72. Woloshuk CP, Sisler HD, Vigil EL. Action of the antipenetrant, tricyclazole, on appressoria of *Pyricularia oryzae*. Physiol Plant Pathol. 1983; 22(2): 245–59. [doi: 10.1016/S0048-4059(83)81013-3](https://doi.org/10.1016/S0048-4059(83)81013-3)
73. Magar PB, Acharya B, Pandey B. Use of chemical fungicides for the management of Rice Blast (*Pyricularia grisea*) disease at Jyotinagar, Chitwan, Nepal. Int J Appl Sci Biotechnol. 2015; 3(3): 474. doi: 10.3126/ijasbt.v3i3.13287
