# Supplementary material for: Oligomycins inhibit Magnaporthe oryzae Triticum and suppress wheat blast disease
Source: PLoS One. 2020 Aug 17;15(8):e0233665. doi: 10.1371/journal.pone.0233665 (PMC7430738; doi:10.1371/journal.pone.0233665)
Supplement: Attachment — Submitted filename: Manuscript editorial comments.docx [file pone.0233665.s002.docx]

**Note that my page numbers below will refer to the page numbers on the edited copy (where editorial comments were inserted) as opposed to the original.**

Line:

1: Title; the term pathotype has been used by other researchers for this taxon but it is not officially accepted; currently there is no formal acceptance of the taxon *Magnaporthe oryzae triticum* but this is evolving nomenclature*.*  In any case I would not capitalize triticum. When taxa include the genera of plants in the binomial (or trinomial), they are not capitalized.

14: “…and the oomycete *Plasmopara viticola.*” The reference that this comes from only indicates activity against this particularl oomycete, and you cannot assume all “peronosporomyctes” are equally affected. The author of the cited paper took excessive liberties in using the term peronosporomycetes.

21: omit “disease” here and in other places where it follows “wheat blast”. Wheat blast is a disease so we don’t have to indicate that it is a disease.

22: while the term “mycelia” continues to crop up in recent manuscripts, I am not in favor of its use. Mycelium is plural, so why pluralize it again? “Mycelia” may be appropriate when you are speaking of mycelium from several different isolates or a collection, like “mycelia of Basidiomycetes”. You occasionally use the term “mycelium”, be consistent and use mycelium throughout.

30: “process of conidial formation” I don’t know what this means; state specifically what process you are talking about.

95: “Most of the synthetic fungicides are harmful…” This is an overstatement and certainly references cited (25-26) would not support the statement since that was not the nature of their research. These references may make that claim but repeating the claim would not be acceptable since these research papers were not about demonstrating fungicides are harmful to humans.

100: “Nowadays, natural products…” This is a spurious argument considering the fungicides that are used for wheat blast have an LD 50 that are well more than twice as high as the LD 50’s of oligomycins. It’s not good enough to simply state that natural products are safer than synthetic ones. It is interesting to note that strobilurins are analogs of natural products.

105: While this statement may be generally true, you are implying that this is true for oligomycins and there is no evidence that this is the case.

131: “A pure culture of a single colony…” this is an odd statement; perhaps you meant, “A pure culture from a single spore.”

136: The references cited, 14-29 do not refer to using 500 ml water to remove aerial mycelium.

150: perhaps the ethyl acetate should have been a control as well.

150: You need to state the “active ingredient” (a.i.) of these materials. Are the oligomycines 100% pure? The fungicide is not; see <https://www.cropscience.bayer.co.za/en/Products/Fungicides/Nativo.aspx>. I believe that the fungicide is 10 and 20% a.i. for the two components.

151: state the volume of material that was loaded into the discs

155: you should state the space difference between the paper disc and the mycelial plug.

156-158: Rather than call Nativo a positive control, I would refer to it as the “industry standard”.

159: Earlier you referred to Nativo as a positive control and here you refer to it as a negative control.

163: This is unclear. I think you mean that each treatment, and the controls were done in duplicate and the experiment was repeated five times.

164: Did you measure the radius or the diameter; most people measure the diameter. I think the term radial is ok but it’s not clear what you measured.

165: Since the colonies were not circular, you need to indicate where you took the measurements, in the middle of the colony? In the widest place? You measured in two directions, did you take the average? When I measure the growth in the figures in millimeters and did the math according to your equation, I came up with very different numbers. I probably measured differently than you.

170: To make these observations, did you make microscope mounts on slides? If from the petri dish did you observe through the top or the bottom of the dish? Did you use oil with the 100X lens?

170-172: “Hyphal morphology at the vicinity of compounds was observed…” and “…and images of morphological features of hyphae were recorded…” This needs to be clarified. I think you are saying. “Hyphal morphology at the leading edge of the colonies facing the treated and control discs were observed with a Zeiss Primo Star microscope at 40X and 100X. (you need to say how you did this, for example 100x is an oil emersion lens). Macro images of the disc diffusion experiment were captured with a Canon DOS 700D digital camera. Images of the hyphae were captured with a Zeiss Axiocam ERc 5s through the microscope.” I don’t know if I am describing your work correctly.

184: Early you stated that washing the cultures was to remove aerial mycelium and here you say it was to reduce nutrients. And, the same references are used. I do not believe either reference is appropriate for this technique. Please re-read these references to be sure. Also, you need to be consistent as to why you washed the cultures.

189: If you are going to say “optimum humidity and lights” then it implies that you know the humidity and light and you should express what it is; otherwise don’t mention it was optimum.

210: Did you pipette the conidia onto a microscope slide and use a coverglass and oil-emersion? You need to explain exactly how you carried this out. The quality of the images in Figure 6 do not look like they were taken at 100X under oil. Also, it is troubling that the field of view shown varies considerably with regard to spore concentration. Spore germination can be affected by the concentration of spores. I realize the concentration was the same, but the Figure 6 shows few spores. It would have been better to selectively image the various stages (shown in Table 1) under oil with high quality images.

230: Describe the plates

232: Did you inoculate 3 leaves for each treatment?

233: Was the leaf where the solution was placed, dry after 15 minutes?

234: 1 × 10^5^ conidia/ml? or per 1 microliter?

268: Why is there a reference to Dame et al.? Are the figures from a paper of his?

269: and Figure 2a**:** It appears that the inoculum and the control disc were placed next to each other; please explain

308: I don’t see what is being described by “…destruction of regular growth with twisted ridges and corrugations, and irregular swelling of hyphal cells.” You must use some other descriptors; I can see differences but not twisted ridges and corrugations. Also, I do not see swelling of hyphal cells in the figures. Did you measure them?

310: I don’t see the crystals in the Nativo figure, or the twisted ridges and corrugations. I am not sure I can see “loss of polar growth”. How does it grow without polar growth?

323: Figure 5. I can’t tell much from these figures. It is hard to justify them if they do not show what they need to show. Perhaps my copies are not very good.

366: Do you mean that after the first 6 hours of germination, 14.3% had lysed? If so, I would write. “…and 14.3± 0.7% of the germ tubes lysed within the first 6 hr of incubation.”

365 and 371 and Figure 6: Does “short” mean shorter than the control? Or does it mean normal? If it was shorter than the control, add “…shorter than the control”. You might add “approximately by half or a third”, or some other figure. Also, for figure 6, it would have been helpful to point out normal, short, long and appressoria in the figures. Unfortunately, the figures are of poor quality and do not show very well the differences. Since the counts and statistics were done on the observations, it was not necessary to take random pictures of the microscopic field; would have been better to selectively take images at higher magnifications illustrating the above differences in morphology.

379-380: “…but a slower growth of germ tubes…” This is the same thing as “short germ tubes”, right? If so, use the same descriptions for all three treatments.

382: It is not necessary to have (*Mot*) in the figure text since you will not refer to it again in the figure text.

388-399: The subtitle as written is biased (as are some of the others in the results); it emphasizes the oligiomycins when the experiments are a comparison of a fungicide with the oligomycins.

407 (Fig. 7) It needs to be indicated on the figure which leaves are represented by 5, 10 and 100 milligrams. You may also mention that “a representative leaf of five replicates”

423-427: Redundant sentences

435: It may be over-reaching to use the term “potential” without considering the possible economic cost of the application. Perhaps a bit of math may shed some light on this.

439: Were these studies actually on the *development* of plant disease protection products?

444: I cannot see any evidence of swelling in Fig 2b and 2c. This should have been quantified by measurements and imaging of single hyphae. In the figure, where hyphae appear to be wider, it could easily be an artifact of the hyphae being close together. It should be a simple matter to treat some colonies, do the measurements and take new images of hyphae.

445: this statement requires a reference or two.

446: what would have been the micrograms per ml?

447: no data has been presented to show that increasing conc. Increases the dia of the hyphae

466: it is not clear that reduced conidiogenesis would occur on wheat leaves.

472: you need to add arrows to the Fig to help this statement

482: No data was presented to support this statement. There may be a different was of bringing this idea forward.
